# Multisystem physiological perspective of human frailty and its modulation by physical activity

**DOI:** 10.1152/physrev.00037.2021

**Published:** 2022-10-14

**Authors:** Joseph A. Taylor, Paul L. Greenhaff, David B. Bartlett, Thomas A. Jackson, Niharika A. Duggal, Janet M. Lord

**Affiliations:** ^1^MRC-Versus Arthritis Centre for Musculoskeletal Ageing Research, School of Life Sciences, University of Nottingham, Queen’s Medical Centre, Nottingham, United Kingdom; ^2^NIHR Nottingham Biomedical Research Centre, University of Nottingham, Queen’s Medical Centre, Nottingham, United Kingdom; ^3^Division of Medical Oncology, Department of Medicine, Duke University, Durham, North Carolina; ^4^Department of Nutritional Sciences, Faculty of Health and Medical Sciences, University of Surrey, Guildford, United Kingdom; ^5^MRC-Versus Arthritis Centre for Musculoskeletal Ageing Research, Institute of Inflammation and Ageing, University of Birmingham, Birmingham, United Kingdom; ^6^NIHR Birmingham Biomedical Research Centre, University Hospital Birmingham and University of Birmingham, Birmingham, United Kingdom

**Keywords:** brain, cardiovascular system, exercise, frailty, inflammation

## Abstract

“Frailty” is a term used to refer to a state characterized by enhanced vulnerability to, and impaired recovery from, stressors compared with a nonfrail state, which is increasingly viewed as a loss of resilience. With increasing life expectancy and the associated rise in years spent with physical frailty, there is a need to understand the clinical and physiological features of frailty and the factors driving it. We describe the clinical definitions of age-related frailty and their limitations in allowing us to understand the pathogenesis of this prevalent condition. Given that age-related frailty manifests in the form of functional declines such as poor balance, falls, and immobility, as an alternative we view frailty from a physiological viewpoint and describe what is known of the organ-based components of frailty, including adiposity, the brain, and neuromuscular, skeletal muscle, immune, and cardiovascular systems, as individual systems and as components in multisystem dysregulation. By doing so we aim to highlight current understanding of the physiological phenotype of frailty and reveal key knowledge gaps and potential mechanistic drivers of the trajectory to frailty. We also review the studies in humans that have intervened with exercise to reduce frailty. We conclude that more longitudinal and interventional clinical studies are required in older adults. Such observational studies should interrogate the progression from a nonfrail to a frail state, assessing individual elements of frailty to produce a deep physiological phenotype of the syndrome. The findings will identify mechanistic drivers of frailty and allow targeted interventions to diminish frailty progression.

## Graphical abstract

**Figure:**
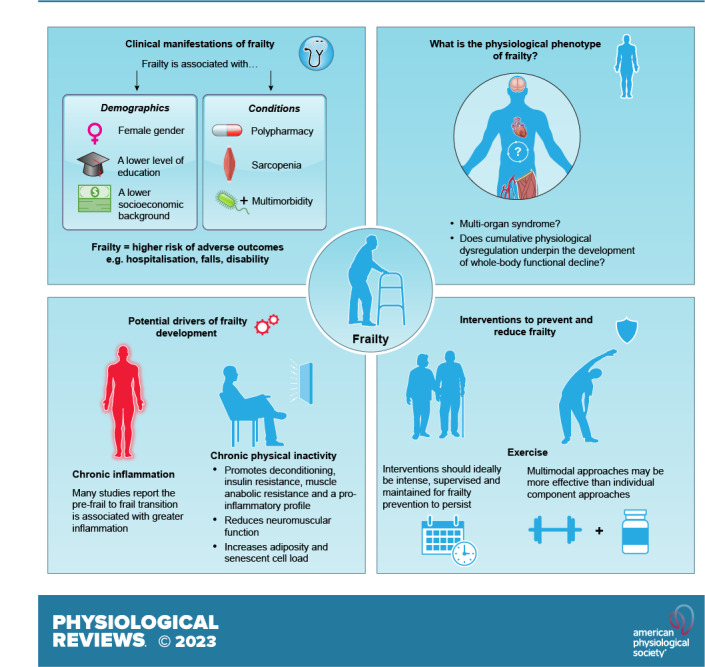

CLINICAL HIGHLIGHTS

- Frailty assessment is currently used as a diagnostic score to estimate risk in older people at times of ill health, such as bed rest, surgery, infections, and bone fractures.
- Clinicians typically use frailty to predict adverse outcomes in older patients, such as risk of dying, good or poor recovery, and moving into a care home.
- Clinicians use multimodal interventions to manage frailty. These have been shown to slow progression of frailty and reverse frailty. As a greater understanding of the underlying physiological dysregulation and biology grows, so should robust trials of new interventions, based on physical activity, nutrition, and pharmacological agents.
- A more detailed physiological systems approach is needed to standardize frailty assessments that will enable clinicians to describe the heterogeneity in health and physical function progression as humans age with greater insight and sensitivity. This will need a multidisciplinary approach involving geriatricians and physiologists employing longitudinal study designs.

## 1. INTRODUCTION

As a result of advances in medicine and public health policy over the last 150 years, life expectancy has doubled and continues to increase globally. In the United Kingdom, one in four adults are predicted to be aged over 65 yr by the year 2050 and 20% of boys and 26% of girls born in 2019 are expected to reach their 100th birthday (1). However, although we are living longer, we are spending more years in ill health, as healthy life expectancy (the length of time we can expect to live in a healthy, disease-free state) has not kept pace with the extension in life span. In the period from 2009–2011 to 2016–2018, life expectancy in the United Kingdom increased by 0.8 yr and 0.6 yr for males and females, respectively. In contrast, healthy life expectancy for males increased by 0.4 yr, and for females it actually decreased by 0.2 yr in the same period (2). As a result of the failure of healthy life expectancy to keep pace with life span extension over decades, older males now spend an average of 16.5 yr in ill health and for women this is 19.8 yr, with multimorbidity and frailty major components of poor health in old age.

Frailty is a largely age-related clinical syndrome characterized by the physiological decline in several body systems, resulting in an increased vulnerability to poor health outcomes and death (3). A systematic review of data from 62 countries, covering >1.7 million individuals, revealed a global prevalence for frailty of between 12% and 24%, dependent upon the specific method for frailty assessment used (4). The transition from health to frailty is a critical factor in the loss of independence in old age. Indeed, the impact on health and social care services of an aging population has led the United Kingdom government to set a target of adults spending 5 more years in independent living by 2035. Understanding the factors influencing the progression to frailty and developing practical approaches to prevent this progression will be key to achieving this target.

In this review, we describe the clinical and physiological features of frailty from an organ/systems-based perspective and the evidence that increased systemic inflammation, increased physical inactivity, and sedentary behavior, with consequent increased adiposity, play roles in frailty development. We review the evidence for the ability of exercise and physical activity to reduce frailty in older adults. We conclude with our perspective on the major knowledge gaps regarding our understanding of the physiology of frailty and priorities for future research.

## 2. THE CLINICAL PHENOTYPE OF FRAILTY

### 2.1. Current Definitions of Frailty

Initial descriptions of frailty tended to describe a static physiological phenotype (5), which was first challenged in the 1990s by Rockwood and colleagues (6), who instead suggested a description of frailty as a dynamic model that balances assets and deficits. This ultimately provided a mathematical framework to describe the heterogeneity of aging, estimating frailty as the difference between biological and chronological age (7). As such, an exercise to describe a typical person with frailty may seem counterintuitive. However, it provides an initial structure for our review from which to explore the physiological phenotype of frailty.

**FIGURE 1.**
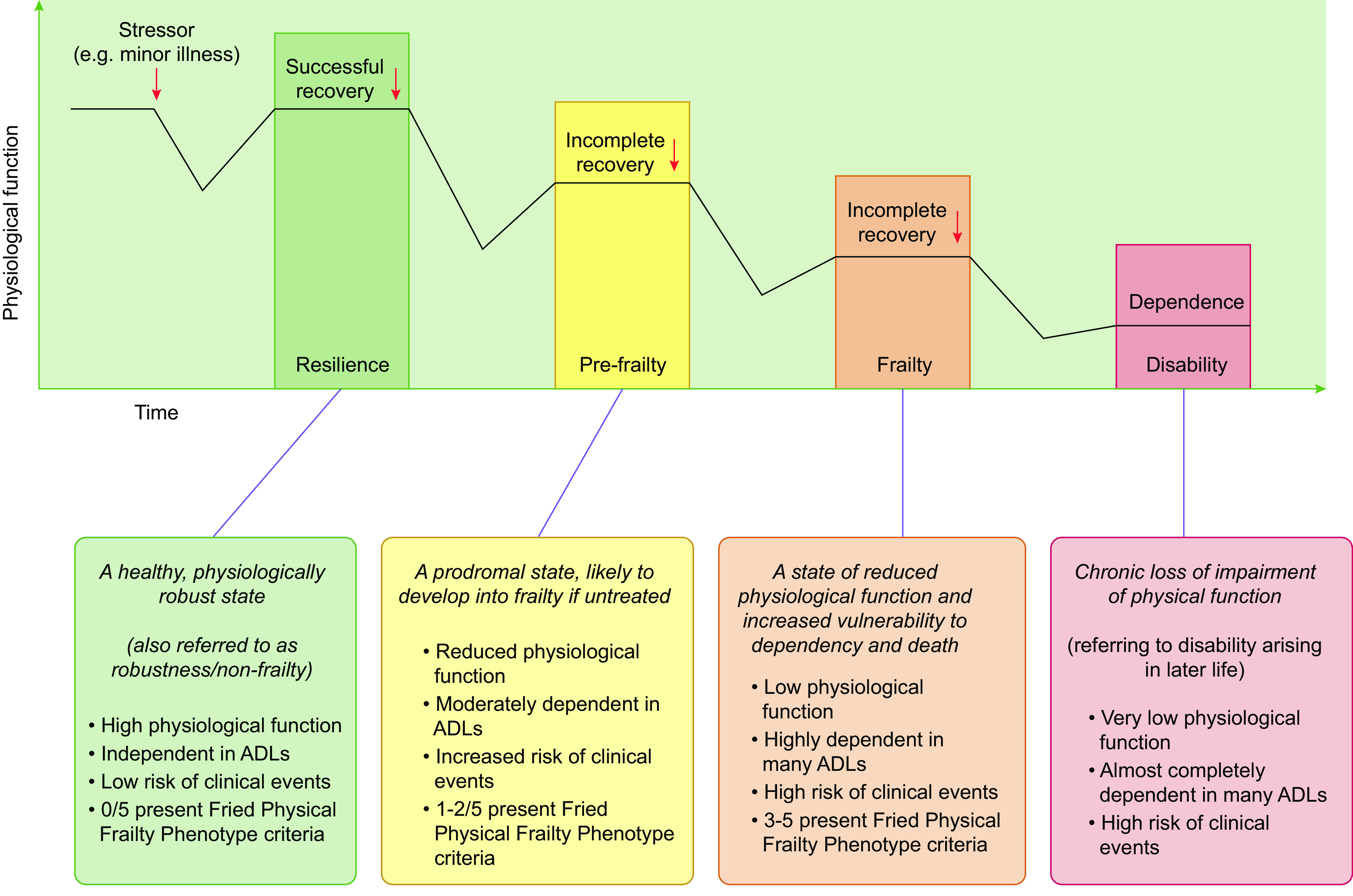
Key stages in the development of frailty. The cascade of functional decline in older adults from an independent (resilient) nonfrail state through to frailty and disability (in the absence of intervention). See glossary for abbreviations. Figure adapted from Ref. 8, with permission under the Creative Commons license: https://creativecommons.org/licenses/by/4.0/.

A consensus group has defined frailty as “a medical syndrome with multiple causes and contributors that is characterized by diminished strength, endurance, and reduced physiologic function that increases an individual’s vulnerability for developing increased dependency and/or death” (3) (FIGURE 1). Importantly, frailty is conceptually different from, but distinctly related to, aging, comorbidity, and disability (9, 10). For example, in a large cross-sectional study of frail individuals, 29.1% of people had an activities of daily living (ADL) disability, and 81.8% had one or more comorbidities (10). These findings underpin the difficulties in producing an exact frailty definition by showing that frailty can present alongside, and potentially be a consequence of, disability and comorbidity but may also occur in the absence of these conditions. The absence of detailed physiological insight pertaining to the condition undoubtedly contributes to the current lack of understanding of frailty etiology and progression.

Despite this lack of understanding, frailty is strongly associated with an increased risk of adverse events, including falls, hospitalization, and mortality (11, 12). Furthermore, some signs and symptoms appear essential for describing the frailty state, the most important of which may be the deterioration of physical function, specifically decreased performance in measures such as skeletal muscle strength, mobility, and ADL, which is highly predictive of frailty presence (13). Conceptually, frailty development involves decreases in functional capacity following a stressor event (e.g., a minor acute illness or fall), with this capacity then remaining at a lower level than baseline after recovery from the event (8) (FIGURE 1), in short, a lack of resilience to return to prior functional capacity. Progressively decreasing functional capacity instigates a cascade of functional decline resulting in frailty, whereby an individual loses independence and becomes at significantly increased risk of disability, morbidity, and mortality (14, 15).

### 2.2. Frailty Assessment

Although usually present, functional decline is not the only clear presentation of a frail individual. Instead, frailty is typically defined by multiple measures of functional decline. Fried and colleagues (16) have operationalized this as the concurrent presence of three or more of the following criteria: low grip strength, slow walking speed, exhaustion, low physical activity levels, and unintentional weight loss. This is termed the physical frailty phenotype; these authors also defined a state of prefrailty, when one or two criteria are present, identifying individuals at increased risk of becoming frail (16). The physical frailty phenotype is currently the recommended international standard for frailty identification and assessment (8). Rockwood and colleagues (17) have used deficit accumulation to determine the presence of frailty by employing a frailty index, which is calculated by considering a number (usually 40 or more) of potential deficits (e.g., age-related symptoms, signs, and diseases). The physical frailty phenotype and frailty index are the two most cited frailty assessment tools within the literature (18), having both been validated as predictive of clinically important outcomes (e.g., hospitalization, mortality) (19).

Because of our lack of knowledge of the underlying pathophysiology of frailty, frailty is currently operationalized by measured outcomes rather than underlying physiological or biological drivers of these outcomes. This lack of consensus of pathophysiology hinders the development of interventions to combat the syndrome’s progression. Therefore, a clear goal for emerging frailty research has been to elucidate the syndrome’s physiological characteristics, enhance knowledge, and improve subsequent treatment options for frail individuals.

### 2.3. Clinical Manifestations of Frailty

Investigations of frailty in human populations commonly describe the proportion of people with frailty within a said population. For example, in a representative survey of 2,740 people aged 65–102 yr from the Canadian Study of Health and Aging, 23% of participants were described as frail by the frailty index definition (17, 20). In a prospective cohort study [the Cardiovascular Health Study (CHS)] that included 5,317 people aged >65 yr but excluded those with dementia, 7% were deemed to be frail by the physical frailty phenotype definition (16). Age was consistently associated with frailty and frailty, therefore, identified in groups of people with age-related diseases, such as 19% of people with COPD and 40% of people with heart failure (21, 22).

**FIGURE 2.**
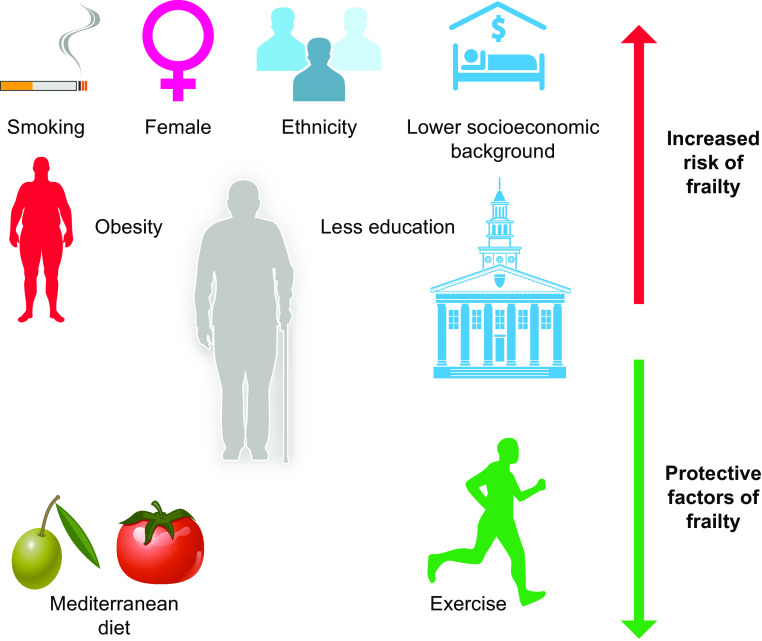
Risk factors for the development of frailty. There are several important risk factors that increase the risk of a person developing frailty. These include sex (female), non-White ethnicity, level of education, socioeconomic status, obesity, and smoking. Protective factors include eating a Mediterranean diet and maintaining physical activity into old age. Image created with BioRender.com, with permission.

Thus, it is also important to consider how a typical person with frailty presents clinically and how frailty affects that person’s individual risks. There are several important risk factors and clinical characteristics identified in longitudinal studies that increase the risk of someone developing frailty over time: People who develop frailty are more likely to be female, of non-White ethnicity, with a lower level of education, and of lower socio-economic backgrounds (23). Clinical risk factors include obesity, depressive symptoms, and smoking. Protective associative factors include eating a Mediterranean diet and maintaining physical activity (23, 24) (FIGURE 2).

**FIGURE 3.**
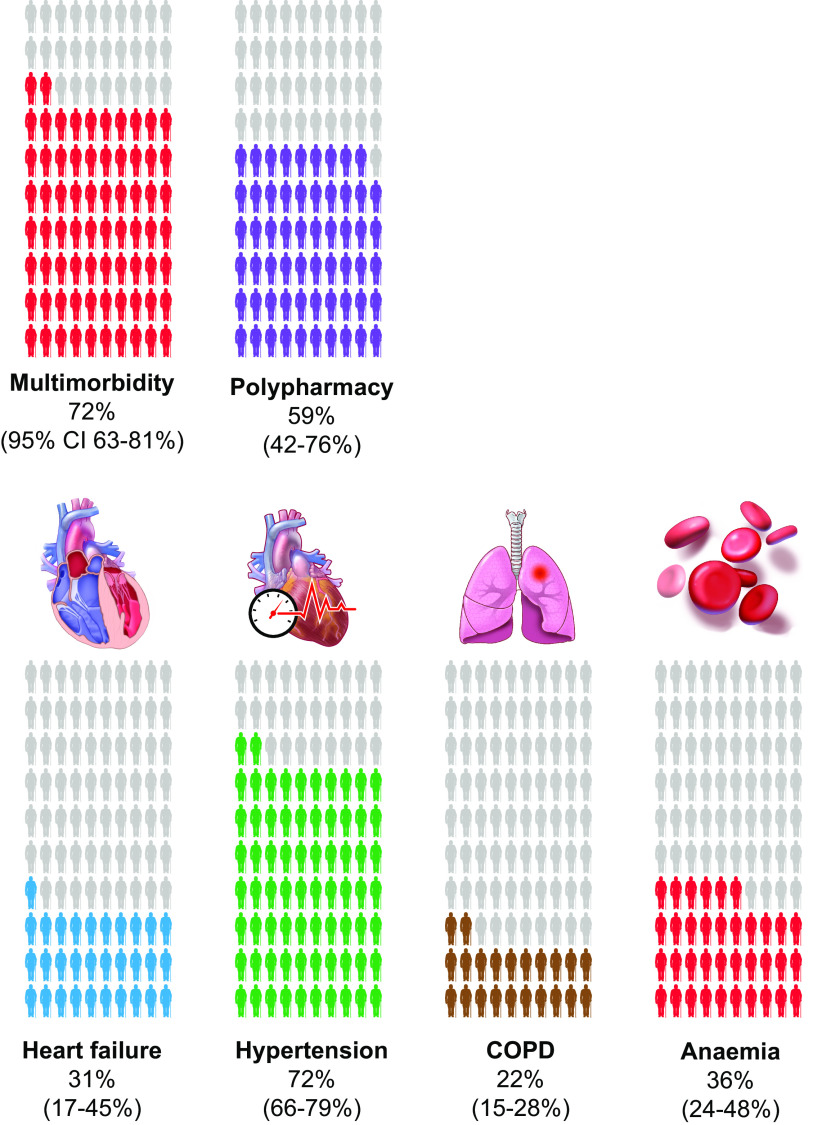
The clinical manifestations of frailty. People with frailty have high rates of heart failure, hypertension, chronic obstructive pulmonary disease (COPD), and anemia. They are also more likely to have multimorbidity (the co-occurrence of 2 or more diseases), polypharmacy, and sarcopenia. CI, confidence interval.

Therefore, our final clinical description of people with frailty identifies common conditions and outcomes associated with aging and reports how commonly people with frailty have them. Frail adults are at higher risk of adverse outcomes, and this is the most important clinical utility of identifying frailty currently. People with frailty are more likely to be hospitalized, fall and fracture bones, and develop a disability, in both physical function and ADL. In addition, people with frailty have high rates of heart failure, cerebrovascular disease, hypertension, COPD, anemia, and diabetes (FIGURE 3). They are also more likely to have multimorbidity (the co-occurrence of 2 or more diseases), polypharmacy, and sarcopenia (TABLE 1). As such, compared with individuals without frailty, people with frailty have a greater risk of death (25).

**Table 1.** Summary of systematic reviews and studies examining the prevalence of age-related conditions in people with frailty

Study	Condition	Study Characteristics	OR of Frailty In People with Condition (95% CI)	OR of Condition in People with Frailty (95% CI)	% of Patients with Frailty Who Have Condition (95% CI)
*Systematic reviews*						
Marengoni et al. 2020 (22)	Heart failure	20 studies in meta-analysis	3.44 (0.75–15.7)		31 (17–45)
Palmer et al. 2019 (46)	Cerebrovascular disease*	18 studies	2.32 (2.11–2.55)		10 (6–13)
Palmer et al. 2019 (47)	Polypharmacy	18 studies in meta-analysis	1.59 (0.90–2.83)	2.62 (1.81–3.79)	59 (42–76)
Vetrano et al. 2018 (79)	Hypertension	27 studies	1.33 (0.94–1.89)		72 (66–79)
Palmer et al. 2018 (83)	Anemia	12 studies in meta-analysis	2.24 (1.53–3.30)		36 (24–48)
Marengoni et al. 2018 (21)	COPD	6 studies in meta-analysis	1.97 (1.53–2.53)		22 (15–28)
Vetrano et al. 2019 (84)	Multimorbidity	25 studies in meta-analysis	2.27 (1.97–2.62)		72 (63–81)
*Individual studies*						
Davies et al. 2018 (85)	Sarcopenia EWGSOP criteria†	Toledo Study of Healthy Aging community based, Spain, >65 yr; *N* = 1,611	1.67 (0.95–2.96)		40.1
	Sarcopenia FNIH criteria‡		10.61 (5.8–19.4)		72.2
Avila-Funes et al. 2009 (86)	Cognitive impairment (lowest quintile)	Community based, Spain >65 yr; *N* = 6,030		1.14 (0.58–2.21)	21.9
Armstrong et al. 2010 (87)	Dementia	23,952 home care recipients, Canada			40.0

*All studies included stroke only. †European Working Group on Sarcopenia in Older People (EWGSOP) algorithm. ‡Foundation for the National Institutes Of Health Biomarkers Consortium Sarcopenia Project. Systematic reviews included here were selected using search terms for frailty and each condition run together, and those that reported a prevalence of each condition in people with frailty with estimated confidence intervals (CIs) were selected. The most recent review was selected if there were >1. OR, odds ratio. See glossary for other abbreviations.

Some diseases are difficult to diagnose in people with frailty if functional impairments from frailty affect the disease itself. Dementia is a clear example, where it is likely that in moderate to severe dementia frailty may well be ubiquitous because of functional and physical impairment caused by dementia. There are positive associations with dementia (26) and worse cognitive impairment in people as the degree of frailty worsens (27). Therefore, dementia highlights how treating frailty as a binary condition, simply present or absent, has limitations. Consideration of the severity of frailty states may begin to lead to more explicit phenotypic definitions of frailty as well as mechanistic understanding of its pathogenesis.

## 3. THE PHYSIOLOGICAL PHENOTYPE OF FRAILTY

The term “phenotype” is defined as “the observable traits of the organism,” covering various characteristics such as morphology, physiology, and behavior (28). The physiological phenotype of the human can be influenced and altered by disease and degenerative syndromes, resulting in measurable distinctions between healthy and disordered states. For example, the condition of sarcopenia, defined as the loss of skeletal muscle mass, quality, and function with age (29), can negatively influence the physiological phenotype of a person through various mechanisms of skeletal muscle deterioration, which leads to observable presentations such as functional decline. Determining exactly how states of health and disorder differ will help identify biological targets for interventions and treatments to combat medical conditions and provide greater insight into the etiology and pathophysiology of complex conditions such as frailty. For example, detailed molecular analyses at the transcriptome level in frailty are now beginning to emerge, including from blood cells and relevant tissues such as skeletal muscle. Zhang et al. analyzed blood cell transcriptomic data for nonagenarians from the Vitality 90+ longitudinal study of aging, comparing nonfrail and frail participants. They identified three genes associated with the emergence of frailty, *TSIX*, *BEST1*, and *ADAMTSL4*, suggestive of key roles for inflammation and regulation of cellular metabolism in frailty, discussed further in sect. 3.2.1 (31). Analysis of the same data set for transcriptomic signatures associated with mortality revealed NF-κB signaling as a key node, reinforcing inflammation as a potential pathophysiological mechanism in frailty (31). Another study has examined the transcriptome of skeletal muscle from healthy young adults and nonfrail and a mixed prefrail and frail group of older adults. Although the differences in gene expression were less marked than between the young and old groups, significant differences were seen between the nonfrail and (pre)frail elders, including for genes regulating muscle function (*MYLK4*) and metabolism (*NNMT*) (32). Importantly, whether these relatively small differences in *MYLK4* and *NNMT* are a driver or a consequence of emerging frailty is unknown but needs to be resolved. Although such transcriptomic analyses may help in mechanistic understanding of the drivers of frailty and aid drug development, perhaps more pertinent, given that people with frailty are invariably at increased risk of adverse events, identifying a distinct physiological phenotype differentiating frail from nonfrail states would be a key priority. Comprehensively characterizing the frailty phenotype would undoubtedly aid in developing strategically targeted interventions against the condition by highlighting typical locations and features of dysregulation.

### 3.1. The Physiological Phenotype of Frailty: the Resting-State Condition

**FIGURE 4.**
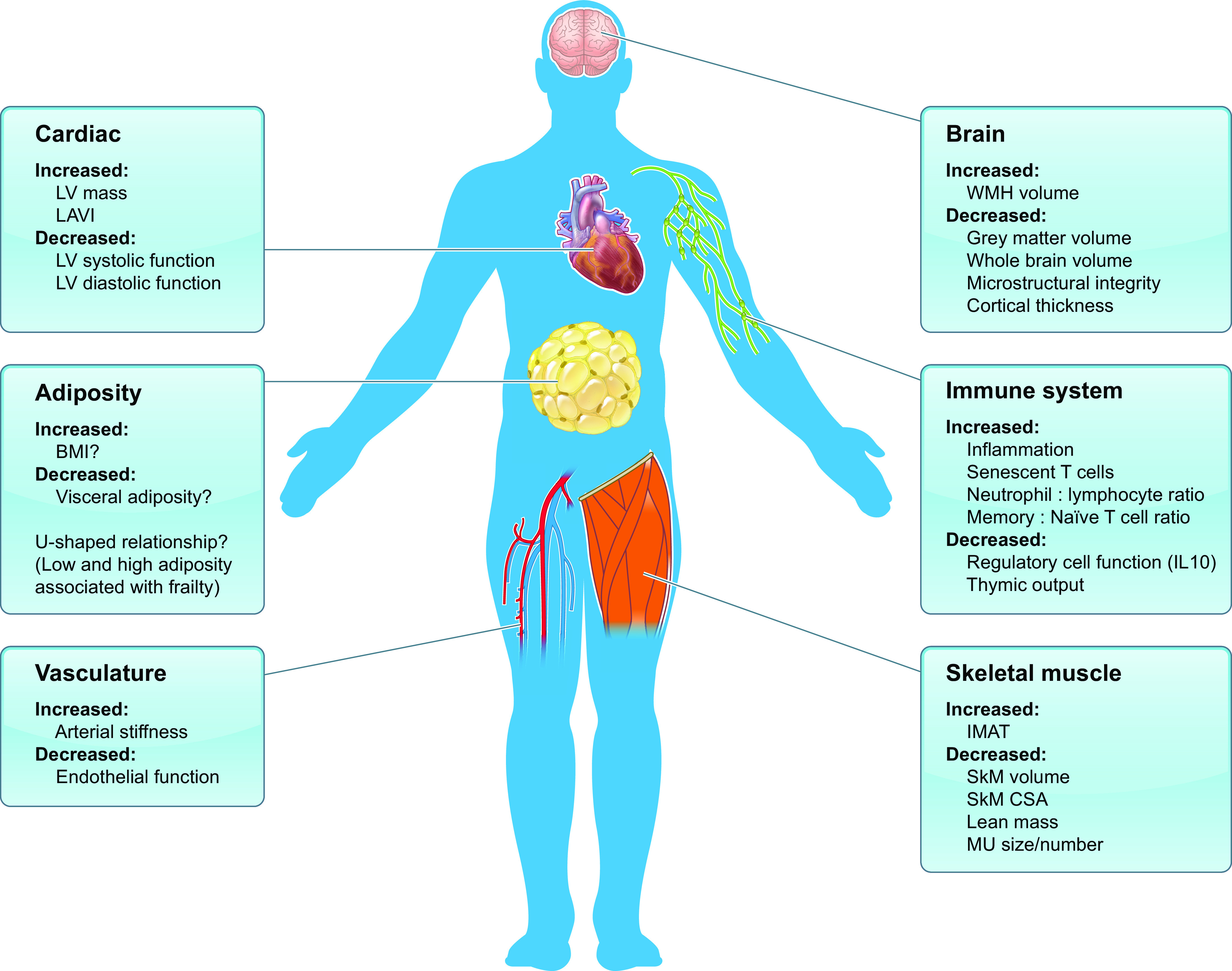
Summary of the typical physiological characteristics of a frail person based on a systems physiology approach. BMI, body mass index; CSA, cross-sectional area; IL-10, interleukin 10; IMAT, intramuscular adipose tissue; LAVI, left atrial volume index; LV, left ventricular; MU, motor unit; SkM, skeletal muscle; WMH, white matter hyperintensity. Image created with BioRender.com, with permission.

Determining the physiological phenotype of human frailty is a challenging prospect. In this way, phenotyping requires intuitive methods to encapsulate complex physiological variables and investigations into how different physiological processes interact and affect each other. In the ideal scenario, the most robust science would require integrative modeling of individual component parts to predict the overall collective response, i.e., the physiological phenotype. However, although the research focus on frailty has increased in recent years, this level of insight is far from being achieved. The majority of studies have involved assessing the physiological characteristics of individual organs under resting-state conditions, which in itself is somewhat incongruous given that frailty seems to be best characterized by a decline in physical functioning and adverse response to stressors. Here we review six systems that contribute in different ways to the frail physiological phenotype, namely, skeletal muscle, the neuromuscular junction and motor unit, the brain, immune and cardiovascular systems, and adiposity (FIGURE 4), and then consider multisystem dysregulation.

#### 3.1.1. Skeletal muscle.

Aging is accompanied by a loss of skeletal muscle mass (33), which often culminates in sarcopenia (29, 34). Sarcopenia reduces insulin sensitivity (35) and is accompanied by deconditioning and the associated loss of mitochondrial mass (36). These observations point to age-related changes in lifestyle factors (e.g., physical inactivity) inducing these muscle-level changes, particularly as prescribed, supervised exercise intervention can at least partly restore muscle mass and function (37) and mitochondrial mass (38), even in frail very old people (39).

Sarcopenia influences functional deficits associated with frailty, including a loss of mobility, decreased strength, and an increased risk of bone fractures (40–42). Therefore, attenuation of skeletal muscle mass and quality likely contributes to frailty development. Frailty and sarcopenia are linked, but distinct, correlates of musculoskeletal aging. This is evidenced by overlap, but incomplete concurrence, in frailty and sarcopenia prevalence (43). Nonetheless, the interrelated nature of frailty and sarcopenia makes it essential to consider skeletal muscle characteristics as contributing factors toward the frailty phenotype (FIGURE 4).

##### 3.1.1.1. whole body lean mass.

Dual-energy X-ray absorptiometry (DEXA) is an X-ray scanning modality allowing the quantification of lean tissue mass (a composite of nonfat and nonbone tissue) and fat mass at a whole body level or regionally. Similarly, bioelectrical impedance analysis (BIA) assesses lean and fat masses based on the notion that lipid-rich adipose tissue is more resistant to the passage of an electrical current compared with tissues rich in water (e.g., muscle tissue). Although DEXA and BIA do not provide direct measures of muscle mass, they are routinely employed in studies of aging, with lean tissue mass observed to decrease with advancing age (so-called sarcopenia) (44). Furthermore, lean mass reductions with age are associated with decreased physical function and quality of life (29, 45) and can be used as a predictor of mortality (48), justifying the use of this parameter as a valid physiological variable. Of published longitudinal studies, Koster et al. (49) reported that the loss of leg lean muscle mass occurred at a rate of 0.7–0.8% per annum during a 7-yr follow-up of individuals in their 70s. In agreement, Frontera et al. (50) demonstrated a 1% per annum decline in thigh muscle mass volume over the course of a 12-yr longitudinal study and concluded that this was a major contributor to the decrease in muscle strength seen over this time. Furthermore, in a cross-sectional study of 18- to 88-yr-old men and women, muscle mass loss was reported to be greater in the lower body, being twice as high as in the upper body (33).

In studies defining frailty with the Fried physical frailty phenotype (16), estimates of lean mass by DEXA revealed a lower whole body lean mass in prefrail and frail people compared with nonfrail people. Furthermore, significant differences were apparent when comparing frail versus prefrail individuals (51). In a study of 1,839 older Taiwanese adults, frail participants had significantly lower total lean body and appendicular lean mass compared with prefrail and nonfrail adults (52). Similarly, whole body lean mass determined by BIA in 220 older adults was significantly less in frail and prefrail compared with nonfrail older males and females (53). However, others have contradicted these findings, reporting no differences in appendicular lean mass across nonfrail, prefrail, and frail subgroups of 250 older women (54).

As outlined above, DEXA and BIA do not quantify muscle mass per se, which adds to the variance in study outcomes focused on muscle mass. To address this issue, advances in mass spectrometry technology have enabled machine sensitivity to be increased, such that orally administered stable-isotope tracers can now be applied to quantify muscle mass directly in community-dwelling people, e.g., the deuterated creatine (D_3_-creatine) dilution method (55–57). This method is based on the assumption that ∼98% of the total body creatine pool is present in skeletal muscle and is turned over in muscle in a nonenzymatic reaction that degrades creatine to creatinine at a constant rate of ∼2 g/day. The additional assumption is that an orally consumed trace amount of D_3_‐creatine has 100% bioavailablity and once absorbed is sequestered by muscle. The urinary excretion of creatine and creatinine and enrichment with D_3_‐creatine allows the muscle enrichment of D_3_‐creatine to be calculated, allowing the determination of the dilution of the tracer in the muscle creatine pool. Of note, the measurement does not require invasive procedures but simply collection of urine and saliva so it could be readily employed in large population studies. This method of assessing skeletal muscle mass in longitudinal large-scale cohort studies may reveal sarcopenia as a powerful biomarker of frailty progression. For example, D_3_‐creatine estimation of muscle mass was associated with functional capacity and risk of injurious falls and disability, whereas assessments of lean body mass or appendicular lean mass by DXA were only weakly or not associated with these outcomes (56).

##### 3.1.1.2. skeletal muscle volume and cross-sectional area.

Quantity of skeletal muscle can also be determined with measures of muscle volume and cross-sectional area (CSA). Magnetic resonance imaging (MRI) and computed tomography (CT) are imaging methods considered the gold standard for muscle volume and CSA measurement, because of their excellent accuracy compared with cadaver analysis (*r *=* *0.99) (58), with these methods utilized to demonstrate muscle volume and CSA reductions in older compared to younger adults (59, 60).

There are few studies utilizing these imaging methods to quantify muscle volume, with CSA being used in most studies of muscle quantity in frailty. A study of 26 older adults reported 6.4% lower thigh muscle CSA in frail compared with nonfrail males and females when quantified by MRI (61). Similarly, MRI-derived average quadriceps muscle CSA of frail hemodialysis patients was lower than that of nonfrail counterparts (62). Comparisons across these studies are hindered by the adoption of different frailty classification criteria. Muscle CSA estimates derived from CT scanning also point to lower skeletal muscle quantity in frailty. In a study of 923 participants, frail adults had significantly lower muscle calf areas compared to those without frailty, albeit numerically small absolute differences (63). A reduced thigh muscle CSA in frail compared with nonfrail nonagenarians has been reported with CT scanning, providing one of the few absolute measures of muscle CSA in frail nonagenarians (64). It should be noted, however, that lower skeletal muscle CSA is not always reported in frail versus nonfrail individuals. For example, one study assessing thigh muscle CSA by MRI observed similar values when comparing nonfrail (*n* = 12) and frail (*n* = 11) individuals (65). The smaller number of frail individuals studied alongside the mixed-sex sample adopted may explain the difference in findings between this study and others. Nonetheless, these discrepancies clearly demonstrate the need for further research to delineate differences in skeletal muscle mass between frailty states. In addition, data derived from imaging methods are needed to definitively illustrate skeletal muscle characteristics evident during frailty, so that key mediators can be targeted with future interventions (e.g., exercise training). For example, if regional differences in muscle volume are apparent during frailty, areas more prone to mass and quality attenuation would be prime targets for interventions.

##### 3.1.1.3. skeletal muscle quality.

It is worth noting that skeletal muscle quantity (i.e., CSA or volume) may not be the only important variable related to muscle within the context of frailty. Recent evidence from multicomponent exercise trials highlights an improvement in functional capacity in older adults, but these gains were not mediated by changes in lower extremity muscle CSA (66). The enhancement of functional capacity evidenced in this study may be attributable to increases in cardiorespiratory function (aerobic capacity) and improved muscle quality, e.g., increased mitochondrial mass, which is consistent with the physiological impact of endurance exercise training intervention in older people (38, 67).

Muscle quality can be assessed from its structural and functional properties, such as muscle aerobic capacity, muscle fiber orientation, myosteatosis, and fibrosis. Muscle quality diminishes with age, which is associated with reduced muscle function and mobility (for review see Ref. 40) and frailty (68).

MRI is a noninvasive and accurate method for assessing skeletal muscle quality, but data in frail individuals are scarce. Melville et al. (69) used MR spectroscopy to highlight greater mean intramuscular adipose tissue (IMAT) content in the vastus lateralis and medialis of prefrail and frail individuals compared with nonfrail counterparts. Although the clustering of prefrail and frail participants into a single group for analysis potentially reduced contrast between groups in this study (69), increased IMAT in the frail has also been reported by others using MRI methods. Addison et al. (61) reported significantly greater IMAT in the thigh muscles of frail compared with nonfrail males and females. Similar findings were also observed in a study utilizing T2-weighted MR imaging, in which frail individuals had a greater intramuscular fat fraction compared with nonfrail subjects (65). Overall, the limited number of studies assessing IMAT support an apparent lipid infiltration of skeletal muscle during frailty. However, generalization of these findings may be hindered by a lack of study power and stratification between sexes (61, 65), given the reported differences in IMAT between older males and females (70).

##### 3.1.1.4. potential drivers and mechanisms of skeletal muscle deterioration in frailty.

Several interconnected and age-related mechanisms potentially contribute to the reported lower skeletal muscle mass, quality, and function in frailty (for reviews see Refs. 71–73). Sarcopenia is considered by many as a core component of frailty (74), with this notion supported by reports of overlap in the presence of sarcopenia and frailty (43). However, definitive longitudinal data in humans are missing.

###### 3.1.1.4.1. Anabolic resistance.

One mechanism proposed to influence the loss of muscle mass in old age is anabolic resistance, the inability of feeding and/or exercise to stimulate muscle protein synthesis or inhibit muscle protein breakdown to the same extent as that seen in young individuals. Seminal research in this area employed stable isotope tracer infusion methods to determine protein turnover in healthy young and older men in response to essential amino acid infusion, thereby avoiding any age-related impact on gut amino acid absorption (75). The authors reported a blunting of muscle protein synthesis in response to essential amino acids in older compared with young participants. Furthermore, the increase in the phosphorylation status of anabolic signaling proteins thought to regulate muscle protein translation initiation, such as mammalian target of rapamycin (mTOR), was also reduced in the older volunteers in response to essential amino acid infusion, indicating that impaired muscle nutrient sensing rather than nutrient availability was underpinning the reduced muscle protein synthetic response. Similarly, a diminished muscle protein synthetic response was observed after a bout of resistance exercise in older compared with young men, which was accompanied by a blunting of the exercise-induced increase in phosphorylation of anabolic signaling molecules (76). Notably, in a study that quantified muscle protein synthesis over the course of a 6-wk resistance exercise intervention, it was observed that chronic muscle protein synthesis was diminished in healthy older compared with young volunteers (77). Furthermore, this was accompanied by a blunted muscle hypertrophic response to the training intervention in the older volunteers, which appeared to reflect blunted ribosomal biogenesis and translational efficiency and lower blood anabolic hormone concentrations (77). It is not known whether the extent of anabolic resistance is greater in older frail adults compared with nonfrail older adults or whether anabolic resistance is a feature of aging per se and/or occurs secondary to factors that accompany aging such as decreased physical activity levels. Nevertheless, the consensus is that deficits in muscle protein synthesis, rather than increases in muscle protein breakdown, is the primary driver of anabolic resistance in older people (78).

###### 3.1.1.4.2. Inflammation.

The vastus lateralis muscle of nonobese frail individuals has been reported to have increased interleukin (IL)-6 mRNA and protein content compared with nonfrail individuals, purportedly because of the release of proinflammatory cytokines from elevated intramuscular adipose tissue in the frail individuals (61). The authors concluded that this intramuscular adipose tissue-inflammatory axis provided a potential link between intramuscular adiposity and decreased muscle mass and mobility function in frailty but did not see any parallel associations involving muscle TNF-α. Nevertheless, potential processes underlying inflammation-mediated muscle loss include exacerbation of anabolic resistance by downregulated muscle anabolic signaling. For example, IL-6 infusion into rodent skeletal muscle at levels consistent with chronic inflammation induces muscle atrophy (80). Atrophy was accompanied by a 60% reduction in the phosphorylation of ribosomal S6 kinase, 33% reduction of pSTAT5, and a twofold increase in pSTAT3 (77). This effect is likely mediated through reduced IGF-1, as transgenic overexpression of IL-6 in mice results in reduced serum IGF-1 levels, possibly due to increased proteolysis of IGF-1 binding protein 3 or increased IGF-1 clearance (81). Accordingly, lower serum IGF-1 concentrations have been observed in frail individuals with low relative appendicular skeletal muscle mass (RASM) compared with frail persons with normal RASM (82).

Other emerging evidence suggests that inflammation contributes to sarcopenia by inducing apoptosis in skeletal muscle fibers, with Chen and colleagues (88) reporting the downregulation of miR-532-3p in muscle from sarcopenic adults. This miRNA targets the proapoptotic gene *BAK1* (BCL2 antagonist/killer 1), and the authors showed that this downregulation was inflammation dependent with NFKB1, a subunit of the transcription factor NF-κB able to bind to the promoter region of miR-532-3p and repress its expression (88). A separate study examined the role of long-chain fatty acids (LCFAs), showing that pentadecanoic acid accumulated in human skeletal muscle in sarcopenia (89), with in vitro studies revealing that this LCFA induced the expression of the transcription factor FOXM1 (Forkhead box M1) and several proapoptotic genes including *PUMA* (p53-upregulated modulator of apoptosis) and *Bax* (B cell/lymphoma 2 associated x).

A third underlying mechanism is the increasing levels of TNF-α in the circulation with advancing age. This cytokine induces upregulation of 11-βHSD1 in skeletal muscle, increasing local generation of the catabolic steroid cortisol. Importantly, expression of 11-βHSD1 in muscle increases with age in women and is negatively correlated with hand grip strength (90). Taken together, these findings present possible mechanisms by which inflammation may induce muscle mass loss during frailty, by impairing muscle regeneration and anabolic processes. However, it is unknown whether these muscle-level characteristics are drivers of muscle deterioration in frailty or a consequence of it.

###### 3.1.1.4.3. Physical inactivity.

As evidenced by reduced step counts and increased sedentary behavior in frail people (91–93), physical inactivity is likely to be another important driver of muscle atrophy and impaired muscle quality, possibly by increased muscle anabolic resistance (94). As people age, physical activity levels tend to decline (95), but studies investigating muscle mass and functional decline with age have rarely controlled for differences in physical activity levels across age groupings in cross-sectional studies. Here, data from studies of episodic periods of increased bed rest are informative, and this will likely induce a greater physiological burden than reduced step count (96). Ten days of bed rest has been shown to induce ∼1-kg lean mass loss from the lower extremities and a 16% decline in knee extensor strength in older individuals (97), which was attributed to a 30% reduction in muscle protein synthesis (97). A metanalysis of transcriptomic data from studies of disuse or bed rest (≥7 days) revealed significant increases in transcripts involved in protein ubiquitination, immune signaling, and apoptosis and downregulation of genes involved in mitcohondrial organization and metabolic function (98), some of the pathways also seen in transcriptomics data from studies of frail elders (30). Other research also highlights bed rest-induced reductions in skeletal muscle protein synthesis that may underpin muscle atrophy and functional losses (99, 100). Moreover, the increased burden of bed rest and illness likely explains why hospitalization will transition an older person from the nonfrail to the frail state (12, 101). Whether bed rest induces increased muscle mass loss and functional decline in an already frail person is currently unknown but warrants consideration.

#### 3.1.2. The neuromuscular junction and motor unit.

**FIGURE 5.**
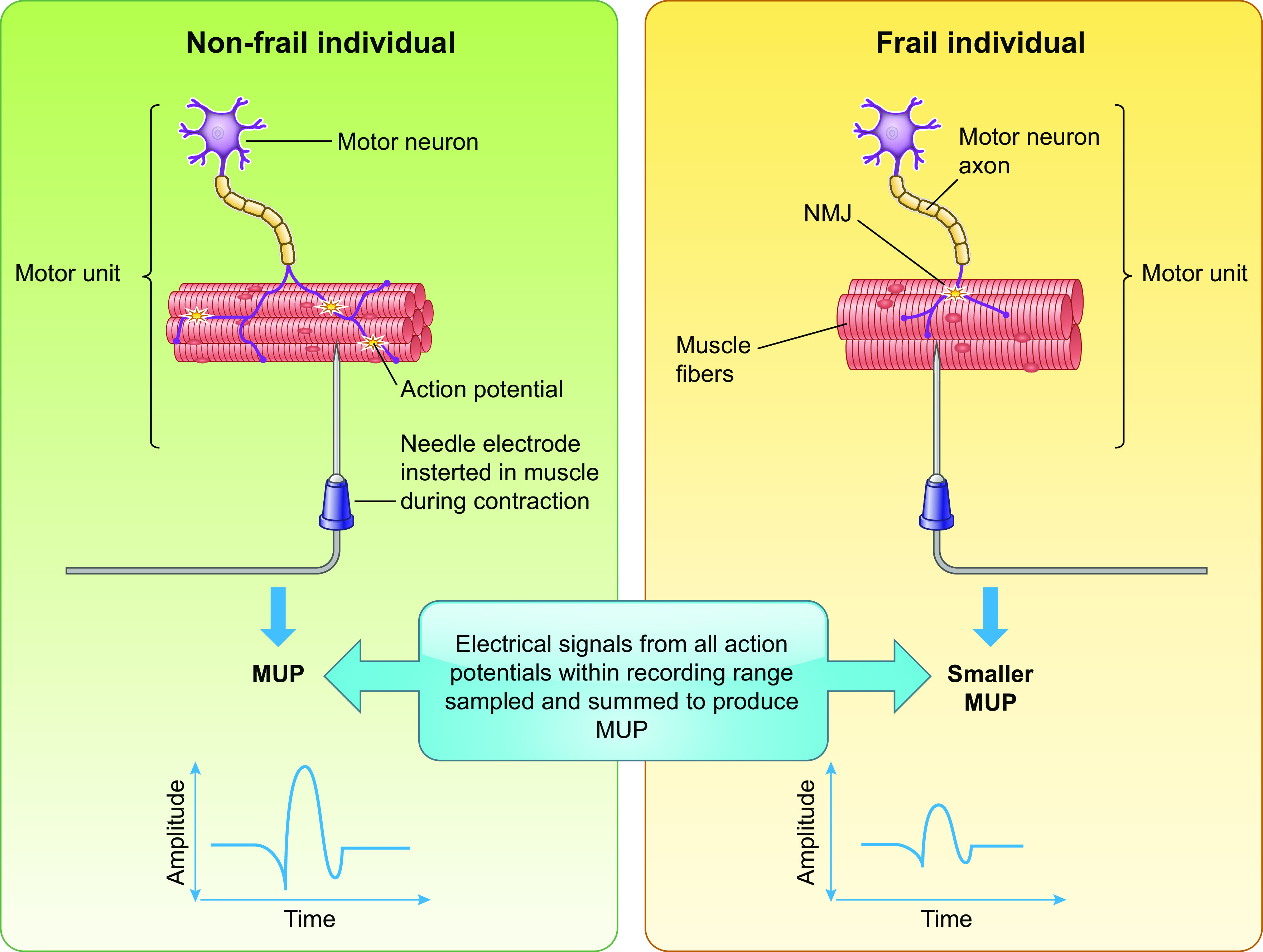
Neuromuscular function in frailty. Schematic overview of the measurement of motor unit potential (MUP) by intramuscular electromyography. Compared to the nonfrail condition, frailty is associated with a smaller MUP thought to arise from smaller motor units. NMJ, neuromuscular junction. Image created with BioRender.com, with permission.

The size and function of the motor unit (MU; the motor neuron and all fibers it innervates) have become a recent focus of aging research, and it has been postulated that muscle fiber atrophy and loss promotes age-related sarcopenia (102). Human MU characteristics can be quantified with the intramuscular electromyography (iEMG) technique. Motor unit potentials (MUPs) (i.e., the sum of action potentials produced by muscle fibers of a motor unit during voluntary contraction) are assessed with this approach, with the size of an MUP proportional to the number of fibers contributing to it (103). Thus, as outlined in FIGURE 5, MUP size is indicative of MU size. Furthermore, a measure of electrical activity termed compound muscle action potential (CMAP) represents a summation of the single-fiber action potentials from all muscle fibers contributing to the signal (104). Dividing the CMAP by the size of an average MUP provides an estimate of the number of MUs within the whole muscle (105).

With advancing age, reorganization of MU fibers is observed [for a comprehensive review of aging effects on the MU and neuromuscular junction (NMJ) see Ref. 106)], which precedes the grouping of fiber types and localized atrophy (107–109). Reorganization includes an increase in MU size with age (110, 111), which is thought to result from branching of nearby motor neurons to reinnervate recently denervated fibers (112, 113). Furthermore, research involving elite master athletes suggests they have a greater capacity to reinnervate muscle fibers (114). Morphological changes also occur at the site of the NMJ, with findings from electron and light microscopy techniques revealing an expansion of the junction perimeter along fibers and more complex branching of the nerve terminal with the synaptic site (115, 116). These morphological changes may occur as an attempt to compensate for a gradual loss of motoneurons during aging as a result of denervation. Indeed, an age-related decline in myelinated neurons has been shown in human peripheral nerves (117, 118), suggesting that aging promotes denervation (FIGURE 5). In conjunction with morphological changes, age-associated neuromuscular deterioration has also been inferred from the lower MU firing rate observed by iEMG in the vastus lateralis of older compared with younger men (111). Furthermore, based on iEMG and muscle cross-sectional area measurements, this study estimated 50–60% fewer MUs in the older participants (111). As well as a reduction in MU number with age (111), it has been proposed that sarcopenic individuals have smaller MUPs during voluntary muscle contractions compared with nonsarcopenic older adults, suggesting that reinnervation of denervated fibers occurs to expand the MU size in the muscle of nonsarcopenic individuals but not during sarcopenia (102). Thus, it is becoming clear that distinct neuromuscular remodeling occurs during aging, alongside sarcopenia, resulting in a reduction in MU number and size.

Building on these findings, increased frailty severity is associated with a smaller size of vastus lateralis MUPs during voluntary contractions and smaller CMAPs generated during electrical stimulation, independent of age and body mass index (BMI) (119). These results suggest that frailty exacerbates MU number and size loss compared with aging without frailty. Given the links between smaller MUs and reduced functional performance (e.g., strength and power) with age (120), the reductions in MU size and number during frailty evidenced by Swiecicka et al. (119) may contribute to the impaired functional performance of the frailty syndrome (68).

##### 3.1.2.1. potential mechanisms for neuromuscular junction and motor unit deterioration during frailty.

As thoroughly reviewed by Larsson and colleagues (106), the mechanisms underlying NMJ and MU deterioration with age are complex and remain poorly understood. DNA damage and modification in old age have been implicated in NMJ functional deterioration and motoneuron loss during aging producing the aged neuromuscular phenotype (122). Spinal motoneurons exhibit apoptotic cell death following treatment with neurotoxic intermediates of glycation, suggesting that by-products of glycation may also contribute to motoneuron degeneration (123). Furthermore, the absence of several molecules involved in NMJ formation and maintenance appears to produce pre- and postsynaptic alterations in aged muscle. Genetic deletion of the molecule agrin (a molecule involved in the formation of synapses between neurons) (124, 125), or its muscle receptor Lrp4 (126, 127), results in degeneration of motor axon terminals and partial or complete denervation of end plates, suggesting that effects on these molecules may contribute to NMJ deterioration (FIGURE 5).

From the perspective of human frailty, the relationship between MU characteristics and plasma concentrations of anabolic hormones has been explored, with free testosterone and dehydroepiandrosterone sulfate (DHEAS) found to be significantly associated with CMAP in frail individuals (121). With the earlier reports of attenuated CMAP in frail men (119), this finding suggests that diminished androgen availability may accelerate MU decline into frailty. Mechanistic insight from a rodent model of spinal cord injury demonstrated that atrophy of motor unit dendrites and muscle fibers was prevented by 4 wk of subcutaneous testosterone administration that maintained normal physiological concentrations (128). Similarly, testosterone administration mitigated motor neuron atrophy following the castration of male adult rats (129, 130). Thus, hypogonadism during frailty may contribute to a decline in MU size and number.

#### 3.1.3. The brain.

**FIGURE 6.**
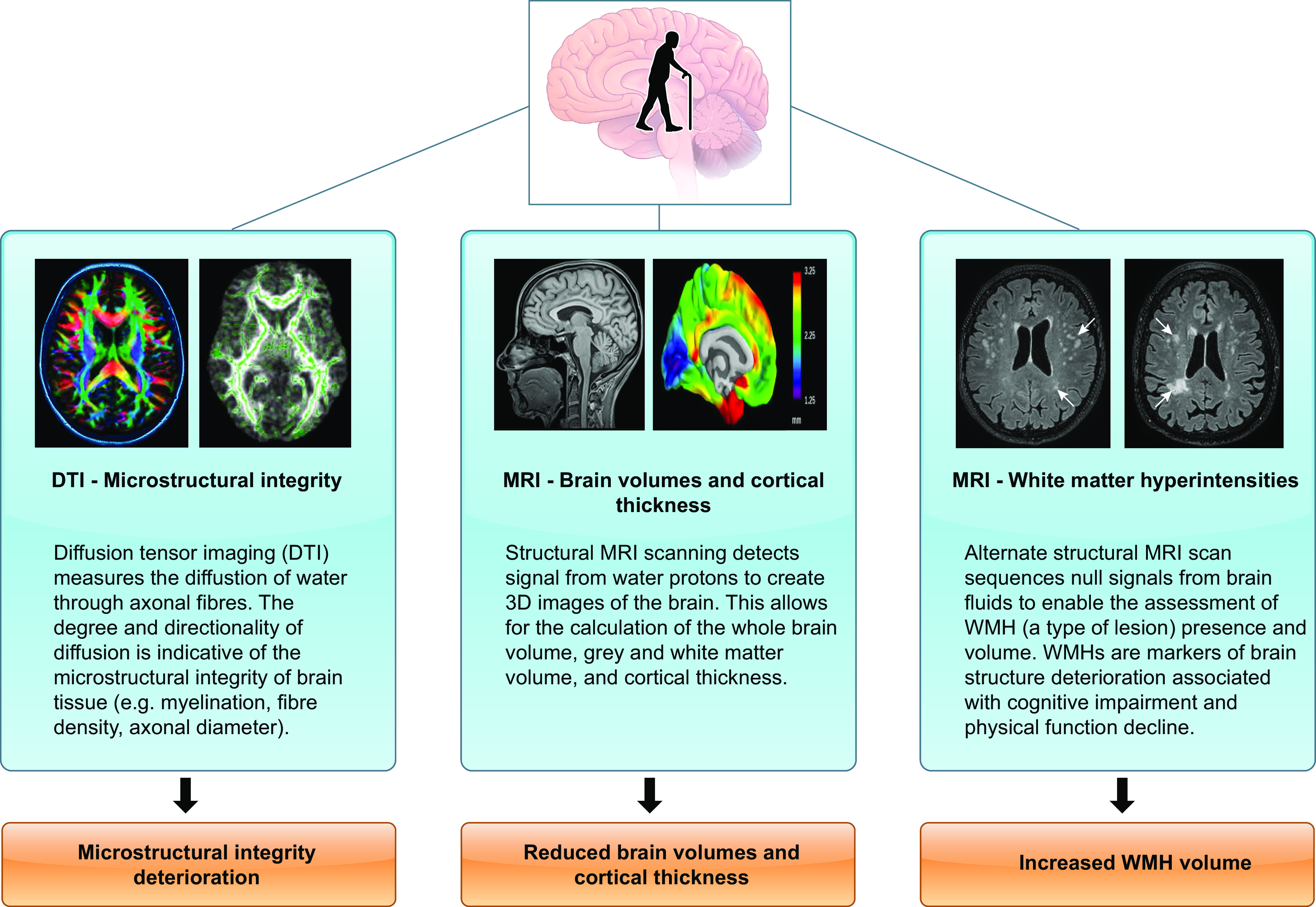
Overview of magnetic resonance imaging (MRI) techniques routinely used to quantify brain architecture in frailty. DTI, diffusion tensor imaging; WMH, white matter hyperintensity. Image created with BioRender.com, with permission.

Aging is associated with various physiological changes in the brain, such as alterations in brain size, vasculature, and cognition (131, 132). Incidence of brain-related diseases such as Alzheimer’s and other dementias also increases with age (133), suggesting that advancing age has profound physiological effects on the brain. Frailty is associated with an increased risk of cognitive decline and dementia (134–136), suggesting that neurodegenerative and neurovascular changes contribute to the physiological phenotype of frailty. Consequently, reported MRI correlates of frailty include lower global or regional brain volume, an increased number of cerebral microbleeds, and a higher number of white matter hyperintensities (WMHs) (134, 137–139). Collectively, these findings provide strong indications of brain structure deterioration during frailty (FIGURE 4) and warrant further investigation of the brain in nonfrail, prefrail, and frail older adults. FIGURE 6 outlines MRI methods currently being employed to study brain architecture and function.

##### 3.1.3.1. brain volume.

Brain volume refers to the mass of nervous tissue within the skull (i.e., the total size of the brain) and can be further partitioned into regional volumes of white matter, gray matter, and cerebrospinal fluid. Measures of total brain volume are strongly correlated with cognitive ability level throughout adulthood (140, 141). During aging, brain volume declines, which is associated with cognitive decline (131, 142) and impairments in physical function (143). Considering the links between frailty, cognitive decline (134, 135), and functional impairments (68), this evidence warrants investigation of brain volumes as key physiological variables during aging and frailty.

Early studies reported global cortical atrophy and reduced gray matter in the brains of frail adults (137, 139). Low recruitment of frail individuals in one of these studies resulted in the combination of prefrail and frail participants into a single group, possibly reducing the contrast between this group and nonfrail adults during analysis (137). Other studies adopting the physical frailty phenotype assessment have provided more detailed findings. Kant et al. (144) reported significantly lower total brain volume and gray matter volume in frail compared with nonfrail older adults. Furthermore, the frail group exhibited lower total brain and gray matter volumes than prefrail participants. No differences were observed between prefrail and nonfrail states (144). Adopting a similar MRI scan sequence, another study also observed total brain volume as significantly reduced in frail versus nonfrail subjects (145). These findings indicate the presence of regional and global brain atrophy during the more severe stages of frailty (FIGURE 6), but again whether associations are causative or a consequence of frailty is not known.

In contrast to these observations, voxel-based analyses of regional gray matter volumes revealed no significant associations between any particular brain region and frailty (146). However, the weakness and slowness criteria of the physical frailty phenotype were associated with reduced gray matter volumes in regions including the hippocampus and the amygdala. Discrepancies with previous research may be attributable to the use of a voxel-based morphometry (VBM) approach as opposed to previous region of interest (ROI)-based methods. VBM involves measurement of tissue volume within each image voxel (or within a specified region), whereas ROI-based methods provide an average estimate of multiple voxels with a large region. This may potentially lead to methodological differences in subsequent image analysis. Nonetheless, these differential findings warrant further research to determine whether frailty per se, or rather elements of the syndrome’s component criteria, are associated with lower brain volumes and in specific brain regions.

Cortical thickness, defined as the distance between the outer cortical surface and the gray-white matter boundary (147), is another structural marker of gray matter volume quantified by MRI (FIGURE 6). Thinning of the cortex in specific brain regions has been shown during normal aging (147–149) and during Alzheimer’s disease (150) and has been proposed as a biomarker of neurodegeneration (151). As far as we are aware, only two studies have assessed the relationship between cortical thickness and frailty. One study reported lower global cortical thickness in frail compared with prefrail and nonfrail participants. However, these authors did not report any statistical evidence for this finding (144). A more recent cross-sectional analysis found that older adults with greater global cortical thickness were less likely to be prefrail and frail (152). These studies indicate that cortical thinning may present during frailty, but further studies are required to confirm these findings.

##### 3.1.3.2. white matter hyperintensities.

Lesions within brain white matter, termed white matter hyperintensities (WMHs), are common features of the aging brain, with an increase in WMH volume observed with advanced age (153). WMHs are also considered MRI markers of cerebral small vessel disease (cSVD) (154). WMHs are associated with adverse outcomes linked to frailty, such as cognitive impairment (155), slow gait (156), and functional decline (157), indicating that these lesions, in addition to cSVD, may present within the pathophysiology of frailty. Recent studies have attempted to clarify the relationship between WMHs and frailty when defined by the physical frailty phenotype (16). Significantly greater mean WMH volume has been observed in frail and prefrail groups compared with nonfrail participants (145, 158). Unfortunately, analysis of WMH volume between prefrail and frail individuals was lacking in these studies, limiting insight between these two states and the progression to frailty. The association of increased WMH volume during frailty has been corroborated in several studies adopting the accumulated deficits frailty index assessment (17), with larger WMH volume shown to be related to higher frailty index scores (159, 160). Furthermore, higher frailty index score has been significantly associated with the presence of mild, moderate, and severe deep WMH and severe periventricular WMH burden (161). Interestingly, with the use of WMH segmentation techniques, it has also been reported that prefrail, but not frail, individuals had a more complex shape of periventricular (situated around ventricles in the brain) and confluent (lesions that extend from a ventricle to >10 mm into deep white matter) WMHs than nonfrail subjects (158). These early reports present an interesting area for further research regarding frailty progression, highlighting WMHs as key markers of brain deterioration during frailty.

##### 3.1.3.3. microstructural integrity.

Diffusion tensor imaging (DTI) is an MRI technique enabling assessment of the microstructural integrity of white and gray matter tissue by mapping the directionality of water molecule diffusion (162) (FIGURE 6). Common measures of diffusion assessed during DTI include fractional anisotropy (FA) and mean diffusivity (MD). DTI has been utilized to demonstrate deterioration in brain microstructural integrity during aging, such as an increase in MD (163, 164), warranting investigation as a physiological feature of the frailty state.

Frail individuals have been observed to have higher MD (indicating degeneration of the tissue that prevents undirected water diffusion) and lower FA in white matter tissue compared with nonfrail counterparts (165), with similar findings also reported in the gray matter tissue of another cohort of frail and nonfrail individuals (145). Furthermore, baseline white matter diffusivity estimates have been significantly associated with worsening frailty over a 5-yr follow-up (166). Common findings of reduced FA and increased MD indicate that frailty is accompanied by degeneration in structural brain tissue through a loss of organized structure.

Some additional findings from these DTI-based studies are noteworthy. First, during region-specific analyses of MD, the medial frontal and anterior cingulate cortexes were strongly associated with frailty (145). The medial frontal cortex is a brain region important for motor function and lower extremity performance, whereas the anterior cingulate is associated with locomotion and gait performance (167–169). These findings suggest that microstructural deterioration in these brain regions may present a physiological cause of functional decline experienced by frail individuals. Second, in frail subjects a larger global WMH volume was associated with decreased FA and increased values in all diffusivity estimates (165). This finding suggests that different features of brain deterioration are linked and negatively influence each other, thereby increasing the risk of frailty development.

##### 3.1.3.4. cerebral perfusion and oxygenation.

**FIGURE 7.**
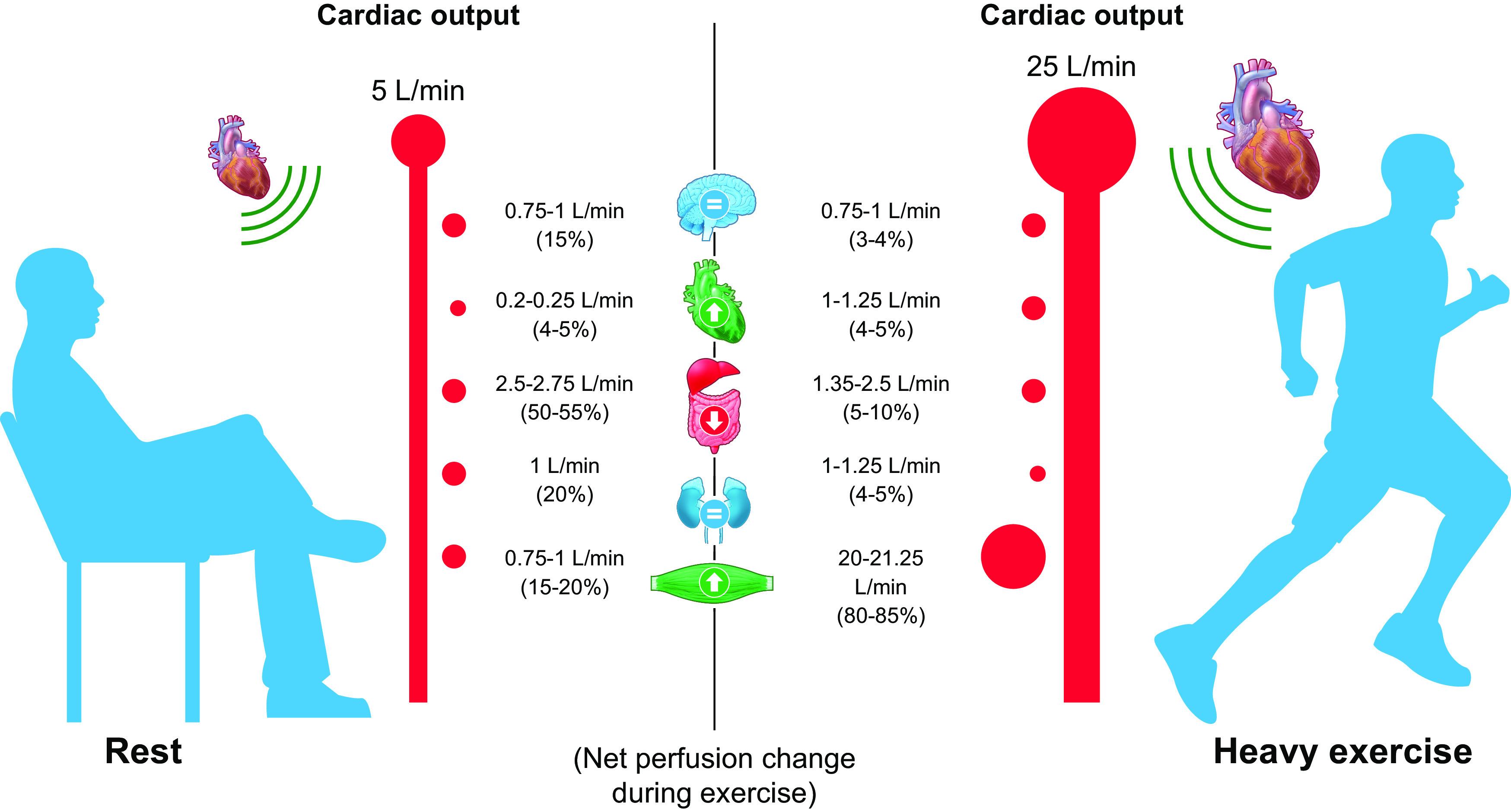
Schematic representation of increased cardiac output and the redistribution of blood flow across organs during exercise compared with rest. Image created with BioRender.com, with permission.

The brain oxygen requirement in the adult human accounts for ∼15% of the resting cardiac output (FIGURE 7), for a relative body size of only 2%. Cerebral perfusion is therefore a high-flow, low-pressure system, which can be quantified with imaging techniques (e.g., MRI and CT). Arterial spin labeling (ASL) is an MRI technique enabling quantification of cerebral perfusion by applying magnetism to “label” arterial blood before flowing into the brain and then subsequently imaging the contrast between labeled blood and brain tissue. Similar to ASL, MRI techniques quantifying cerebral oxygenation can magnetically label venous blood, and the rate at which the magnetic signal is lost is indicative of blood oxygen levels. Cerebral oxygenation can also be quantified by near-infrared spectroscopy (NIRS) and is based on the differential light absorbance of oxyhemoglobin and deoxyhemoglobin, as these “chromophores” absorb different wavelengths of light. Both cerebral perfusion and oxygenation are observed to decline with age (170, 171), and this decline is associated with Alzheimer’s disease and other dementias (172, 173), suggesting that these variables are key physiological markers of neurodegeneration. One study has assessed global gray matter perfusion with ASL, evidencing no association between global gray matter perfusion and frailty (158). This lack of relationship may have been due to the reduced sample size adopted when performing the ASL scanning procedures, which the authors acknowledged compromised the statistical power of their analyses (158). Cerebral oxygenation was previously measured in frail hospital patients during anesthesia by NIRS (174). These authors found increased cerebral desaturation in the frail compared with the nonfrail group, suggesting that oxygenation of the brain is impaired during the frailty state.

##### 3.1.3.5. potential mechanisms of brain deterioration in frailty.

Although current research into brain deterioration during frailty is mainly observational, some insight into potential interrelated mechanisms of brain degeneration can be inferred. One possible mechanism is based on the finding of reduced cerebral perfusion within WMHs (175). Considering this finding, and the higher WMH burden evident during frailty (158, 159), cerebral perfusion may be attenuated. Accordingly, in healthy and cognitively impaired participants relationships between reduced cerebral blood flow and brain atrophy have been observed (176, 177). Furthermore, in a study of middle-aged adults lower cerebral blood flow has been associated with increased brain atrophy, but only in patients with moderate to severe WMH volume burden (178). Taken together, this evidence suggests that WMH-mediated attenuations in cerebral perfusion may contribute to brain deterioration during frailty. However, mechanistic insight cannot be inferred given that the evidence presented in these human studies is only associative. Experimental evidence for the role of reduced cerebral blood flow in the pathogenesis of brain atrophy is provided by animal models (179). However, only one underpowered estimation of cerebral perfusion exists within the human frailty literature (158), leaving this notion speculative at present.

Physical inactivity and increased sedentary behavior have also been conveyed as factors contributing to altered brain structure during aging (180, 181). For example, a recent study demonstrated that a 5-yr decrease in white matter volume was associated with increased amounts of sedentary behavior and reduced physical activity levels when measured by accelerometry methods in nonfrail older adults (182). A previous review outlines evidence to suggest that sedentary behavior and reduced physical activity may cause detrimental effects in the brain through mechanisms such as reduced neurogenesis, synaptic plasticity, and angiogenesis and by increased inflammation (183). Collectively, these findings indicate that physical activity levels and sedentary behavior may mediate the mechanisms leading to reduced total brain volumes (144) and increased WMH volumes (158) in frail individuals.

Neuroinflammation is a common feature of aging (184, 185) and neurodegenerative diseases such as Alzheimer’s disease, Parkinson’s disease, and multiple sclerosis (186). Considering that frailty is an age-related syndrome associated with neurodegenerative disease (187), it seems logical that neuroinflammation may contribute to brain deterioration in frail individuals. However, neuroinflammation has not been explored extensively within the context of frailty. Nevertheless, research combining cerebrospinal fluid sampling and brain MRI indicates that reduced cognitive function is associated with increased levels of the neuroinflammatory marker YKL-40 in older adults (188), with a second 2-yr longitudinal study reporting increased cerebrospinal fluid YKL-40 concentrations associated with loss of microstructural integrity and brain atrophy of older individuals (189). These markers of structural decline are also evident in frailty (145), suggesting that neuroinflammation may contribute to brain deterioration during the syndrome, which warrants further investigation.

Mechanisms of cerebral degeneration are difficult to uncover in human research because of the invasiveness of accessing and sampling brain tissue. However, insight into causal mechanisms may benefit from region-specific analyses when studying the brain in human imaging studies. In the context of frailty, these analyses are helpful, as they may provide specific targets for further research aiming to uncover underlying mechanisms of brain deterioration. For example, during frailty attenuation in brain volume (137, 190) and microstructural integrity (145) has been found within regions of the brain related to physical function, such as the medial frontal and anterior cingulate cortexes. This information could be used in animal models of frailty [e.g., the IL-10 knockout mouse model of frailty (191)] to inform on the mechanistic links between brain deterioration and functional decline during frailty. Alternatively, to provide further insight into human frailty, future studies should adopt protocols similar to Tian et al. (145), where multiple features of brain structure, including brain volumes, WMHs and DTI parameters, are investigated simultaneously. Although this application of multiparametric MRI is not a new approach in human studies, and may even be considered standard practice in Alzheimer’s and dementia research (192, 193), we stress the importance of employing this approach in future frailty work to aid in understanding how different features of brain deterioration interact and potentially exacerbate frailty development.

#### 3.1.4. The cardiovascular system.

The prevalence of cardiovascular disease increases with age (194, 195) and encompasses complex pathophysiology in numerous interrelated organs and tissues. A meta-analysis of 6,000 nonfrail, 7,000 prefrail, and 1,500 frail individuals revealed that frail (odds ratio = 3.4) and prefrail (odds ratio = 1.5) persons are at increased risk of cardiovascular disease compared with nonfrail counterparts (196). This provides associative evidence for the role of cardiovascular dysfunction in the development of frailty. However, the specific alterations in cardiovascular structure and function that might contribute to frailty remain unclear. A summary of cardiac and vascular characteristics present during frailty is shown in FIGURE 4.

##### 3.1.4.1. cardiac parameters.

Aging is associated with various physiological changes in heart structure and function, such as an increase in left ventricular (LV) wall thickness, atrial fibrillation, and a decrease in LV ejection fraction (197). Impairments in cardiac structure and function, assessed by echocardiography, are associated with physical function decline in older individuals (198, 199), suggesting that cardiac dysregulation may contribute to frailty. Some common findings are evident across studies assessing cardiac parameters during frailty. In the Cardiovascular Health Study, increased LV mass was observed in frail versus nonfrail participants (200), with several other studies since reporting an increased LV mass index as well as increased left atrial volume index within frail individuals (201–203). Despite some common findings, inconsistencies have been reported for several other cardiac parameters during frailty. For example, LV ejection fraction (EF) has been observed as significantly attenuated in frail versus nonfrail groups in some studies (202, 203) but not others (204, 205). These differential findings may be due to the mean age of participants in some studies being higher (202) and the adoption of differing echocardiographic protocols. It would be worthwhile to build on these echocardiography-derived findings by employing the less patient- and investigator-dependent cardiac MRI methodology (206–208). Furthermore, cardiac MRI enables the assessment of myocardial scarring and diffuse fibrosis (209), which may be a cause of the increased LV mass observed in frail individuals. As such, it appears there are currently no MRI-based measures of cardiac parameters within the literature associated directly with frailty per se, reinforcing the need to apply this modality to enhance understanding in this area.

In a large sample of frail individuals, increased LV hypertrophy, along with impaired LV systolic and diastolic function, has been found in the frail compared with the nonfrail (203). Interestingly, this study reported greater prevalence of abnormal cardiac measures in the frail even after impairments in the pulmonary, renal, hematologic, and adipose systems had been accounted for in the analysis. Furthermore, cardiac abnormalities, such as LV hypertrophy, showed the greatest association with frailty of all the organ systems studied (203). Collectively these findings suggest that heart dysfunction significantly contributes to the physiological frailty phenotype (FIGURE 4).

##### 3.1.4.2. vascular parameters.

Alterations in the physiological characteristics of the human vasculature are also observed with advancing age, such as increased arterial stiffness (210), wall thickness (211), and endothelial dysfunction (e.g., reduced vasodilatory response and nitric oxide bioavailability) (212, 213). Furthermore, vascular dysfunction is associated with sarcopenia, potentially through decreased muscle microperfusion (214) and sedentariness (215), indicating that pathophysiology within the vasculature may contribute to the phenotype of frailty.

However, only a limited number of studies have assessed parameters of vascular structure and function during frailty. Assessing carotid-femoral pulse wave velocity, two large-sample studies, including the Framingham Heart Study, reported an increase in arterial stiffness during frailty (203, 216). Markers of endothelial dysfunction, such as abnormal ankle-brachial index, pulse wave velocity, and low levels of flow-mediated dilation, have also been associated with frailty (217). Furthermore, frailty has been linked to a greater blood concentration of dimethylarginine (218), which is elevated in endothelial dysfunction and is an independent risk factor for major adverse cardiovascular events, and reduced flow-mediated dilation (219, 220). This small number of studies collectively provide some indications of vascular deterioration during frailty.

##### 3.1.4.3. hypertension.

Hypertension is a well-known cardiovascular risk factor associated with aging (221), with blood pressure, particularly systolic pressure, increasing with age (222). Hypertension may contribute to cardiovascular decline by exacerbating endothelial dysfunction (223) and promoting an increase in LV mass (224). Furthermore, traits related to frailty, such as physical function decline and cognitive impairment, are associated with hypertension (225–227), implying that blood pressure is an important parameter to assess in the context of frailty. However, a systematic review and meta-analysis revealed an inconclusive relationship between frailty and hypertension, with cross-sectional and longitudinal studies reporting mixed results (79). Discrepancies may be due in part to the different frailty assessment criteria adopted across cross-sectional studies, which may partially explain why the meta-analysis failed to show any significant associations. The mixed results from longitudinal analyses (79) are in line with the findings of a randomized control trial (RCT) that was unable to show any impact of treatment of hypertension on the onset of frailty (228). However, a possible explanation for these RCT data may be that individuals developing frailty might be more likely to be lost before follow-up, with this selective dropout making it difficult to draw firm conclusions regarding the effect of the treatment on frailty-related outcomes (229). Nonetheless, these mixed results warrant further investigation of the relationship between frailty and hypertension, ideally with large-sample size longitudinal studies.

##### 3.1.4.4. potential mechanisms of cardiovascular dysfunction in frailty.

###### 3.1.4.4.1. Inflammation.

Higher serum inflammatory markers in older individuals are related to features of cardiac dysregulation, such as increased LV hypertrophy and diastolic dysfunction (230). Given that these cardiac abnormalities are also evident during frailty (203), increased inflammation in frail individuals may contribute to cardiac deterioration. Inflammatory cytokines have been proposed as regulators of cardiac dysregulation through several mechanisms. Overexpression of TNF-α in cardiac tissues in mice leads to proteasome dysfunction and accumulation of ubiquitinated proteins in the left ventricle (231), which may be a mechanism contributing to increased LV mass during frailty (200). Similarly, chronic TNF-α overexpression restricted to cardiac tissues reduces the activity of collagenolytic enzymes, resulting in an attenuation of LV dilation (232). These processes may underpin cardiac dysfunction during frailty, mediated by a chronically heightened inflammatory state in the heart.

###### 3.1.4.4.2. Physical inactivity.

Reduced physical activity levels may also contribute to cardiovascular dysfunction during frailty (91). For example, lower LV EF, which has been noted during frailty (202, 203), is associated with reduced physical activity levels in middle-aged adults (233). This may be explained by physical inactivity-induced promotion of cardiac atrophy (234), which in turn attenuates LV function by less contractile tissue being available for contraction. This is supported by findings of marked reductions in the synthesis of cardiac proteins and significant cardiac tissue loss following limb unloading (235). Increased arterial stiffness in frail individuals may also be contributed to by reduced physical activity, given that higher arterial stiffness is observed in older individuals with increased amounts of sedentary time (236). Arterial stiffening may also be influenced by low vascular blood flow during sedentary time, leading to lower endothelial shear stress and impairments in endothelial function (237). For example, low endothelial shear stress is associated with low nitric oxide synthase expression (238), and blocking nitric oxide synthesis increases arterial stiffness in vivo (239).

#### 3.1.5. The immune system.

**FIGURE 8.**
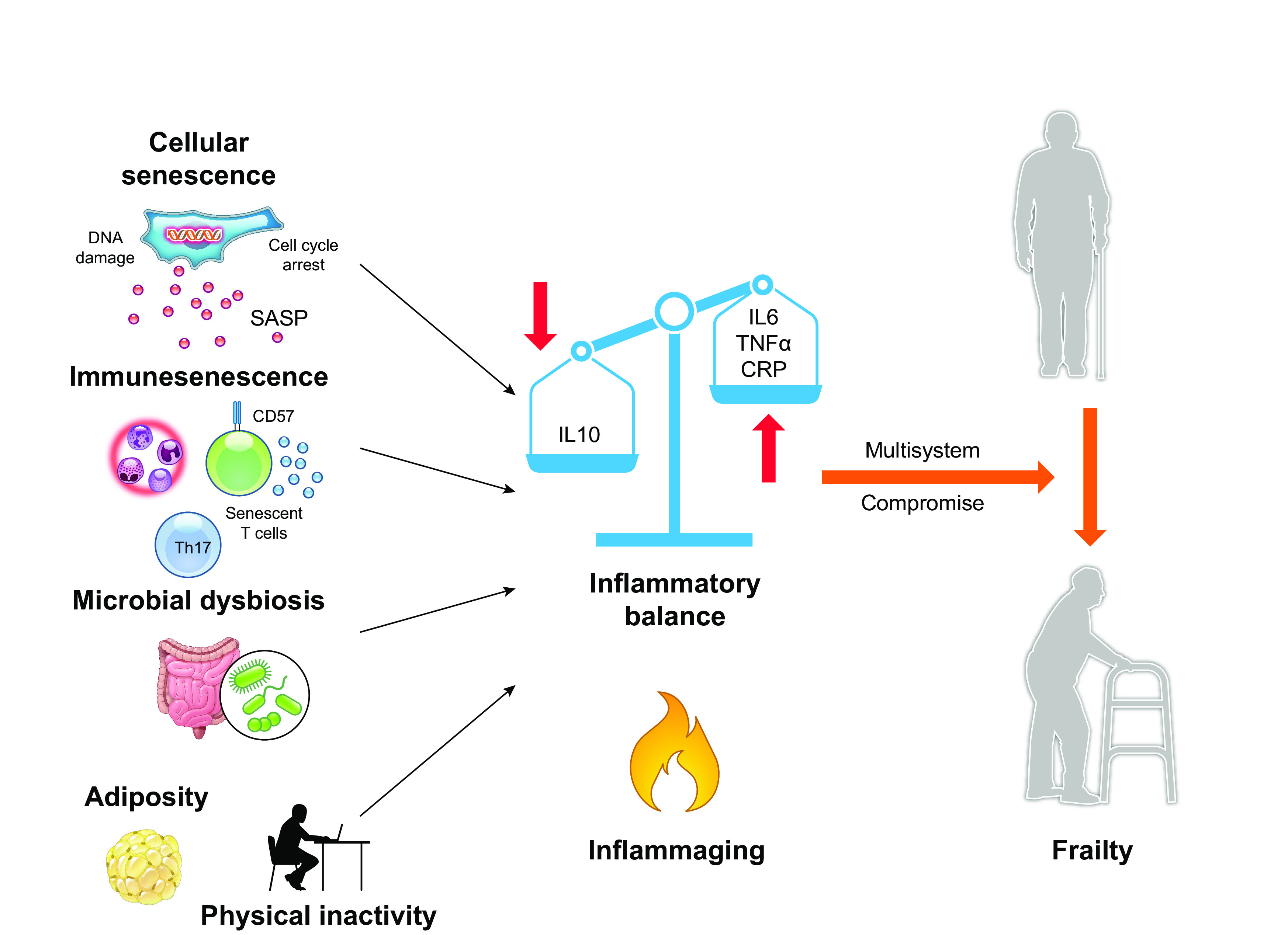
Factors contributing to the age-related increase in systemic inflammation (inflammaging). Increased systemic inflammation with age, inflammaging, is multifactorial in origin. Key contributors include an increase in senescent cells, which have a proinflammatory secretome, the senescence-associated secretory phenotype (SASP); reduced physical activity, which contributes to increased adiposity, with adipose tissue being a source of inflammatory mediators such as adipokines; and gut dysbiosis and reduced intestinal integrity, which lead to leaking of microbes into the circulation that then induces an inflammatory immune response. The degree of inflammaging is associated with increased risk of moving from a nonfrail to a frail state. See glossary for other abbreviations. Image created with BioRender.com, with permission.

As with the four organ systems described above, the immune system is significantly altered with age (FIGURE 4), termed immunesenescence, resulting in a decline in the ability to mount a robust immune response to infection or vaccines and increased risk of autoimmune and chronic inflammatory diseases (240, 241). These age-related changes are also a key factor in the increase in systemic inflammation seen with advancing age, inflammaging (FIGURE 8), which is associated with an increased risk of a broad range of age-related diseases (242). Importantly, the immune system, by the very nature of its function in defending against pathogens, has access to all parts of the body. A compromised immune system thus has the potential to influence functional decline throughout the body and contribute to multisystem dysregulation in frailty. That an aged immune system may have broad influences on organ function and thereby frailty has recently been suggested by studies in mice in which only the T-cell compartment was modified. Specifically, mitochondrial function was compromised by the knockdown of mitochondrial transcription factor A (TFAM), resulting in accelerated T-cell senescence. The TFAM-deficient mice showed an aged phenotype including multimorbidity, reduced physical function, and premature death, a phenotype that was rescued by blocking of TNF-α signaling or restoration of mitochondrial function with nucleoside riboside (243).

As the hallmarks of immunesenescence have been reviewed extensively (244), we focus here on those elements that may support the increased inflammatory status seen in old age and the development of frailty.

##### 3.1.5.1. immunesenescence.

The innate immune system is the first line of defense against pathogens and includes cells such as macrophages. These are tissue-resident sentinel cells that rapidly alert the rest of the immune system to infection by producing inflammatory cytokines. During early life, the innate immune system is able to return to a quiescent state after antigen exposure. However, with advancing age these cells are in a state of low-level constitutive activation resulting in the secretion of proinflammatory cytokines in the absence of infection, contributing to inflammaging (245, 246). The adaptive immune system is also altered with age, driven primarily by the atrophy of the thymus in early adulthood. This results in a reduced production of naive T cells and a consequent expansion of memory T cells to maintain the lymphocyte pool (FIGURE 8). With repeat stimulation across the life course these memory T cells experience telomere attrition and enter a state of terminal differentiation as EMRA (effector memory-expressing RA) cells marked by loss of CD28 and CD27 and expression of CD57 and CD45RA (244). These cells have poor proliferative capacity and are highly proinflammatory, adding to the inflammatory burden (247, 248). Other hallmarks of immunesenescence that contribute to inflammaging include an increased propensity of T cells to differentiate toward the proinflammatory Th1 and Th17 phenotypes (249). Single-cell RNA sequencing has recently identified a subset of age-associated granzyme K-expressing CD8 T cells that amplify the inflammatory phenotype and contribute to inflammaging (250). Furthermore, the immune system has a variety of mechanisms to prevent persistence of an inflammatory state, but these also decline with age. For example, cells including macrophages and regulatory T and B lymphocytes have an anti-inflammatory role, secreting cytokines such as IL-10, but with age their function declines (244, 251), reducing the homeostatic resolution of inflammation. In addition, the immune system plays a key role in removing senescent cells, which are proinflammatory (see below), with natural killer cells and CD8 T cells recognizing these cells via the NKG2D receptor (252). As their cytototoxic ability declines with age this will contribute to the accumulation of senescent cells (253).

That immunesenescence plays a role in frailty in humans is unclear, as few studies have assessed indicators of immune aging in frail and nonfrail individuals and the majority simply compare healthy young and old subjects. However, the Singapore Longitudinal Aging Study assessed markers of T-cell aging in 421 older adults who were nonfrail, prefrail, and frail, showing that loss of CD28 on CD4 and CD8 T cells was positively associated with frailty and CD28-negative CD8 T cells were predictive of a prefrail state (254). A recent 2-yr longitudinal study assessed the neutrophil-to-lymphocyte ratio (NLR) and systemic inflammation index (SII), as indicators of immunesenescence, in 1,822 older adults for their association with incident frailty, using the physical frailty phenotype. Both log NLR and log SII were positively associated with incident frailty; the association remained when adjusted for multimorbidities (255). In contrast, a 5-yr longitudinal study in 657 >85-yr-old subjects found no association of T-cell senescence with loss of muscle function or prevalent or incident sarcopenia (256). Although this study did not report data for frailty, it does support the need for further longitudinal studies and a broad assessment of immunesenescence to identify specific elements that may be contributing to frailty and could be targeted in future interventional studies with compounds such as nucleoside riboside.

##### 3.1.5.2. inflammaging.

Physiological aging is characterized by a chronic state of elevated subclinical levels of proinflammatory cytokines (e.g., TNF-α, IL-6, CRP) termed inflammaging (257). Although the majority of studies of inflammaging do not include measurements of anti-inflammatory cytokines such as IL-10, levels of this cytokine have been reported to decline with age in longitudinal studies (258). It should be noted that other studies have reported a rise in IL-10 with age, suggesting a compensatory mechanism to counterbalance inflammaging (259, 260) (FIGURE 8). This dynamic progression to a proinflammatory state has been recognized as a biomarker of biological aging associated with an increased risk of a broad range of age-related diseases (261). For example, inflammaging has been associated with increased cognitive impairment (262), cardiac dysregulation (230), sarcopenia (263), cancer (264), and Alzheimer’s disease (265). In contrast, studies in centenarians (266) and naturally long-lived mice (267) show a cytokine profile similar to younger people/mice with no inflammaging. Furthermore, even in those who are not among the exceptionally long lived, inflammaging is not an inevitable consequence of advancing age; for example, several studies have shown that maintaining high levels of physical activity into old age will prevent inflammaging (268). Inflammaging is therefore not inevitable and may well be an index of adiposity (see sect. 3.1.6) or an early indicator of biological aging and decline toward frailty.

The majority of studies in humans investigating associations between inflammation and frailty are cross-sectional in nature, with fewer longitudinal studies or clinical trials using anti-inflammatory drugs to test for causality. Nevertheless, indirect support for a causative role of inflammation in frailty can be deduced from the IL-10-deficient mouse, which develops a frail phenotype with many similarities to humans (269), and the IKK2 knockout mouse, which has compromised NF-κB activation and shows preservation of muscle mass (270).

###### 3.1.5.2.1. Cross-sectional studies.

Evidence from multiple cross-sectional studies supports a positive relationship between increased systemic inflammation with age and frailty, some directly assessing frailty but others providing indirect evidence by focusing on elements of sarcopenia (for reviews see Refs. 271–273). Elevated circulating levels of proinflammatory cytokines (e.g., TNF-α, IL-6, CRP) have been associated with loss of muscle mass and strength (274), poor physical performance (275), loss of aerobic fitness (276), and disability (277). Interestingly, studies examining sex-specific differences have observed a stronger association between markers for inflammation and frailty in women than in men, potentially driven by sex differences in body fat quantity and distribution (278). Fried’s multiparameter analysis of systems affected in frail older adults also showed that older women with three or more divergent systems, including inflammation, were more likely to be frail (279).

A systematic review of 50 studies has revealed that several elements of an increased inflammatory status, i.e., raised IL-6, TNF-α, CRP, neopterin, fibrinogen, and neutrophil and monocyte counts, are present in frail adults (280). A 2016 systematic review and meta-analysis of 32 cross-sectional studies also showed that the prefrail and frail states were associated with higher CRP, IL-6, fibrinogen, and leukocyte counts (263). Furthermore, a recent analysis of the plasma proteome to determine biomarkers of frailty in 752 older adults from the InCHIANTI study found that four proteins [creatine kinase M-type, B-type CKB, C-X-C motif chemokine ligand 13 (CXCL13), and thrombospondin 2] were associated with frailty (281). In addition to associations with circulating levels of cytokines, a strong linkage between several single-nucleotide polymorphisms (SNPs) in the *CRP* gene (rs3093059, rs2794520, rs1205) and reduced handgrip strength in older adults has been identified (282). Another study reported that frail individuals carry a CRP (1846G > A) gene polymorphism, an underpinning factor contributing toward elevated frailty (283). Additionally, an inverse correlation has also been observed between the production of proinflammatory cytokines (such as TNF-α) and handgrip strength in older adults (284).

###### 3.1.5.2.2. Longitudinal studies.

Longitudinal studies, though less numerous than cross-sectional studies, have been performed to assess associations between increased blood inflammation status and frailty. A longitudinal study in 901 healthy older adults assessing physical functioning in the participants 9 yr apart reported a significant increase in IL-6 levels and a 21% decline in grip strength and gait speed over the study period (285). Similar longitudinal relationships between higher CRP and lower grip strength have been reported in large-scale birth cohort studies (286). In the InCHIANTI cohort study mentioned above, two proteins, cyclin-dependent kinase 5 and IL-1α, were associated with worsening of frailty in a longitudinal analysis (281), supporting a role of inflammation. A smaller longitudinal study sampled 144 adults from middle age every 5 yr up to 65–75 yr of age. The data revealed that elevated levels of IL-6 pathway markers, namely CRP and sIL-6R, were associated with more frailty and reduced physical strength. Other associations were detected in women, notably increasing sCD14 levels and frailty, an indicator of monocyte overactivation (287). In contrast, in a recent longitudinal study of a large birth cohort (*n* = 1,091) the physical frailty phenotype and frailty index were both used to assess frailty in participants 12 yr apart. They found higher CRP associated with increased frailty at follow-up as assessed by the frailty index but not by the physical frailty phenotype (288). Some of the discrepancies in findings may therefore reflect differences in the frailty assessment used.

###### 3.1.5.2.3. Evidence from anti-inflammatory interventions.

There are few interventional studies using anti-inflammatory drugs in humans with frailty as an end point, with most assessing different aspects of sarcopenia. A systematic review considered 28 studies assessing the impact of anti-inflammatory drugs on inflammation and skeletal muscle. Not all of the studies were in older adults, but those that were found that celecoxib and piroxicam, two nonsteroidal anti-inflammatory drugs, could reduce inflammation and improve physical performance in older adults with raised systemic inflammation. They also found that ibuprofen increased exercise-induced muscle hypertrophy and muscle strength and, in general, concluded that the effects on muscle were achieved most consistently when combined with exercise (289). Pharmacological blockade of IL-6 by tocilizumab and inhibition of Jak/STAT3 pathway by ruxolitinib have been shown to suppress muscle atrophy by downregulating the expression of the atrophy genes *MuRF1* and *MAFbx* in vitro and in an animal atrophy model (290). In addition, senolytic drugs, which remove proinflammatory senescent cells, reduce frailty in mice (291) and improve physical function in humans (292). It is important to point out that the beneficial effects of blocking inflammation for muscle adaptation to exercise may not extend to older adults not exhibiting raised systemic inflammation (293). Although the effects of NSAIDS on muscle protein synthesis have shown mixed results, they have been suggested to compromise satellite cell activity (294).

Taken together, these studies suggest that the emergence of inflammaging is coincident with elevated frailty in humans with age, but further evidence, especially from longitudinal and interventional studies that include the transition from the nonfrail to frail state, is required to support any causal relationship in humans.

##### 3.1.5.3. potential mechanisms contributing to inflammaging.

In addition to the contribution made by immunesenescence, inflammaging is a multifactorial process with a range of genetic (295) and environmental factors identified that contribute toward its development (296) (FIGURE 8).

###### 3.1.5.3.1. Cell senescence.

Cell senescence is a state of irreversible cell cycle arrest induced by various stressors, including DNA damage, telomere shortening, and protein aggregation. Cell senescence has been identified as one of the nine hallmarks of aging that underlie the development of the aged phenotype (297). Removal of these cells, either genetically (298) or pharmacologically through the use of senolytic drugs (299), has been shown to extend life span and healthpan in mice. Trials are now underway in humans with senolytic drugs, the first of which (dasatinib and quercetin) reported improved physical function in patients with idiopathic pulmonary fibrosis (292). Importantly, although senescent cells are proliferatively quiescent, they are highly metabolically active. In particular, they produce a secretome, the senescence-associated secretory phenotype (SASP), containing a broad range of proinflammatory cytokines and chemokines as well as proteases and growth factors. These cells accumulate in the body with age and therefore contribute to inflammaging through their SASP (300).

###### 3.1.5.3.2. Microbial dysbiosis.

Gut microbial composition changes dramatically with advancing age, including a reduced abundance of anti-inflammatory bacterial species (e.g., *Bifidobacterium spp.* and *F. prausnitzii*) and an expansion of proinflammatory pathogenic microbes (e.g., *Streptococcus spp.* and *Staphylococcus spp*.) termed microbial dysbiosis (301). Additionally, the intestinal barrier deteriorates with age, resulting in increased mucosal barrier permeability, allowing translocation of microbes and toxins into the circulation (302), with an associated increase in systemic immune cell activation and inflammation (303, 304). Studies in mice have revealed that cohousing aged mice with young germ-free mice increases systemic inflammation and immunesenescence in the young mice as they ingest feces of the aged mice and acquire their gut microbiome (305). These data together suggest that age-related dysbiosis contributes to immunesenescence and inflammaging, though these findings need to be confirmed in humans.

###### 3.1.5.3.3. Physical inactivity.

A wealth of observational studies have confirmed that regular physical activity is associated with lower levels of circulating proinflammatory cytokines, such as CRP and IL-6 (306, 307). In a recent meta-analysis, data from eight exercise intervention studies (resistance, aerobic, and combined) showed a positive effect of exercise in reducing the inflammatory profile in older adults (308). The potential mechanisms by which physical activity exerts an anti-inflammaging effect include reduction in fat mass; we discuss the potential role of adiposity in inflammaging and frailty further in sect. 3.1.6. Part of the proinflammatory nature of adipose tissue is based upon the infiltration of monocytes/macrophages and senescent cells, which then produce proinflammatory cytokines (309). Studies in mice have shown that enforced physical inactivity (withdrawal of a running wheel) led to an increased senescent cell load in adipose tissue that was prevented by exercise (310). Importantly, exercising muscle is anti-inflammatory. When released from exercising muscle, IL-6 is termed a myokine and, in this context, produces systemic anti-inflammatory effects (311) via a variety of actions including increased levels of anti-inflammatory cytokines IL-10 and IL-1RA as well as cortisol (312). IL-6 is thus a dual-functioning cytokine, with its actions very much context dependent; when produced by immune cells and at a high circulating level, such as during infection, it is proinflammatory, but when produced at lower levels, such as during exercise, it acts on macrophages to switch them to an M2 phenotype producing anti-inflammatory cytokines (313).

#### 3.1.6. Adipose tissue.

Aging is associated with increased adiposity, such as increased whole body and abdominal fat deposition (314–317). This age-related increase in abdominal adiposity is reportedly mainly attributable to increased visceral, as opposed to subcutaneous, fat deposition (318, 319). The health implications of increased adiposity with age are complex and still poorly understood, with adiposity in overweight and obese older people being positively associated with mortality in some studies (320, 321) but not others (322). Being overweight and obese has even been associated with better outcomes in various medical conditions (322–324) and a reduced risk of clinical events in frail individuals (325). Nonetheless, the links between adiposity and physical function deterioration and disability (326, 327), in conjunction with the presence of weight loss as a component criterion of the physical frailty phenotype (16), warrant the investigation of adipose tissue within the context of frailty.

Crude indexes of obesity (e.g., BMI ≥ 30 kg/m^2^ and waist circumference) have been adopted as indirect assessments of adiposity within studies of frailty, producing conflicting results. A systematic review of six longitudinal studies revealed a direct association between obesity and the incidence of frailty (23). For example, a longitudinal study among 28,181 older women reported an almost fourfold-increased risk of developing frailty in obese individuals compared to those with a normal BMI, after a 3-yr follow-up (328). This finding has been confirmed in another large-sample study, showing an increased risk of frailty with each additional year of obesity (329). Cross-sectional data also highlight that obesity is associated with a higher risk of prefrailty and frailty in women aged 70–79 yr (330). Whether this is a direct causative relationship is unknown, but the association remained statistically significant after adjustment for multiple conditions (diabetes mellitus, heart failure, etc.) and inflammation status (330).

In contrast to the above findings, longitudinal studies illustrate that low BMI (<18.5 kg/m^2^) is associated with the risk of frailty compared with normal BMI (18.5–24.9 kg/m^2^) (328). This observation is corroborated by cross-sectional data highlighting a significantly lower BMI in frail versus nonfrail individuals (331). Accordingly, a U-shaped relationship between frailty and adiposity may be evident, with low and high (as opposed to normal) levels of adipose tissue contributing to increased risk of frailty, which would be consistent with BMI data (328). However, the adoption of crude and indirect assessments of adiposity (i.e., body mass and waist circumference) in these studies limits insight into the relationship between frailty and adiposity.

Studies quantifying adiposity with imaging techniques during frailty are rare. Idaote et al. (64) highlighted greater pericardial and visceral adipose tissue in the lumbar region of nonfrail compared with frail older participants after CT scanning, providing support for the longitudinal data highlighting associations between low BMI and frailty (328). Reduced adiposity may therefore underpin the typical nonintentional weight loss trait exhibited by frail persons (16). However, a large-sample study adopting CT scanning observed similar lower leg adipose tissue CSA in nonfrail and frail individuals (63). Direct comparison of the results of this study to those of Idaote et al. (64) is difficult because of differences in quantification of adipose tissue stores in different body regions. Consequently, research in this area would benefit from utilizing imaging techniques to directly quantify whole body and regional adiposity with longitudinal study designs, to better understand the complex relationship between frailty and adipose tissue.

DEXA estimates of fat mass also reveal mixed findings regarding the link between frailty and adiposity, with one study reporting a greater body fat percentage (i.e., total fat mass in relation to total body mass) in frail compared with nonfrail participants (51). However, when expressed as an absolute estimate (measured in grams), the difference in total body fat mass was nonsignificant. DEXA estimates of total fat mass have also been highlighted as similar between nonfrail, prefrail, and frail individuals in a large Taiwanese sample (52) and a smaller cohort from the Women’s Health and Aging Study (54). Thus, these conflicting results underscore the poor understanding of the relationship between frailty and adiposity, reinforcing the requirement for uniform measurement approaches and large-sample longitudinal studies to progress this area.

##### 3.1.6.1. potential mechanisms of altered adiposity during frailty.

Physical inactivity and high levels of sedentary behavior contribute to increased fat mass (332, 333). Considering that these behaviors are associated with frailty (91, 334) and low physical activity is a component criterion of the physical frailty phenotype (16), inactivity may contribute to increased fat mass during the syndrome. Mechanisms mediating physical inactivity-induced elevations in adiposity may include a reduction in skeletal muscle insulin sensitivity, leading to the accumulation of central and visceral adipose tissue (335, 336). For example, bed rest models of inactivity highlight a reduction in insulin sensitivity and dysregulated lipid and glucose oxidation in tandem with increased adiposity and IMAT accumulation (337), particularly under conditions of positive energy balance (338, 339). These findings are reinforced by reports of greater rates of hepatic free fatty acid uptake in individuals with low physical activity levels (340), whereas habitual endurance training is associated with a reduced hepatic free fatty acid uptake (341). Although these findings are not specific to frailty, they present potential mechanisms by which inactivity contributes to increased adiposity in frail individuals.

Increased adiposity may be contributing to the enhanced inflammatory state evident in frail individuals (342, 343). Higher levels of circulating IL-6 have been attributed to increased fat mass and obesity (344), with previous work demonstrating that up to 30% of circulating levels of IL-6 may be released from subcutaneous adipose tissue in obese subjects (345). Proinflammatory cytokines may in turn negatively influence other physiological systems, such as muscle mass and function (274). IMAT is also a proposed site of inflammatory cytokine release. Accordingly, increased IMAT and IL-6 protein content in the vastus lateralis has been observed during frailty (61), perhaps suggesting that larger IMAT stores may further contribute to an enhanced inflammatory environment and facilitate skeletal muscle atrophy in frail individuals. Indeed, obese older men, who presented with heightened systemic inflammation and far greater adiposity compared with their nonobese age-matched counterparts, also experienced a blunting of the acute muscle protein synthetic response to increased nutrient delivery (346). However, these same individuals presented with greater lean tissue mass and had no impairment of muscle strength or work done during repeated knee extensor contractions. Analysis of muscle mRNA expression in these obese older men showed reduced levels of transcripts for cytochrome *c*, peroxisome proliferator-activated receptor-α, peroxisome proliferator-activated receptor-γ coactivator 1α, and TFAM, which are associated with mitochondrial biogenesis or oxidative phosphorylation, whereas expression of myostatin, a negative regulator of muscle growth, was greater in obese skeletal muscle (346). Whether these observations in nonfrail men are representative of frail people is unknown, but the mRNA pattern was consistent with muscle deconditioning being a driver of metabolic dysregulation (346), which is pertinent to frailty. Importantly, it is unknown whether any of these muscle-level characteristics are drivers of muscle deterioration in obesity or a consequence of it.

#### 3.1.7. Multisystem dysregulation.

Research on aging and frailty biomarkers, including most studies cited above, has traditionally focused on individual biomarkers. However, investigations into single-mechanism explanations of aging, such as inflammation and oxidative stress, have produced multifactorial explanations, in which multiple physiological processes interact (347, 348). This has led to the proposal of nine hallmarks of aging, comprising a sequence of processes that lead to the aged phenotype in various organ systems. The sequence is initiated by the accumulation of damage within cells, producing responses such as mitochondrial dysfunction and cell senescence, with end points of inflammation and reduced stem cell turnover effecting biological aging (297). This understanding has led to a change in how aging, and in turn frailty, mechanisms are perceived, with many researchers now acknowledging multisystem physiological dysregulation as a key biological underpinning of health decline during aging.

The rationale for considering frailty as a state of several disordered systems is provided by the links between frailty and different syndromes such as sarcopenia (349), vascular dementia (136), and heart failure (200) (FIGURE 4). Furthermore, results from the Cardiovascular Health Study cohort revealed associations between frailty and dysregulation in the cardiac, vascular, and cerebral systems (200), although in this study these systems were not evaluated together regarding their contribution to the presence of frailty. Nonetheless, collectively these findings point to dysregulation in multiple physiological systems during frailty, which has instigated a focus of research in this area.

Multisystem dysregulation was first investigated by analyzing 12 biomarkers in eight different physiological systems (anemia, inflammation, IGF-1, DHEAS, hemoglobin A1c, micronutrients, adiposity, and fine motor speed) of frail and nonfrail older women (279). It was demonstrated that an increasing number of abnormal physiological systems were related to an increased likelihood of being frail, with abnormality in three or more systems deemed a significant predictor of frailty (279). Notably, the cumulative number of dysregulated systems, as opposed to any specific system, was the dominating factor predicting frailty severity. The relationship between accelerating frailty and an increasing number of abnormal systems was nonlinear (279), suggesting there may be a threshold beyond which an adverse downward spiral of frailty progression is evident. This would be consistent with the concept of “majority rules” in systems biology (350, 351), whereby the aggregate of impaired systems may adversely affect the function of other unimpaired systems driving the whole system to a more dysregulated state.

Frailty at a multisystem level has also been investigated with a statistical approach that estimates physiological dysregulation during aging by assessing the difference between a discrete biomarker value and the average value for a population mean (347). Using data from nearly 33,000 individuals, and analysis of 37 biomarkers grouped into six physiological systems (lipids, immune, oxygen transport, liver function, vitamins, and electrolytes), Li et al. (352) revealed dysregulation in several systems and proposed the establishment of a global dysregulation score (collated estimates on all biomarkers) that predicts the magnitude of frailty presence. Interestingly, no individual system was markedly better at predicting frailty than another (352). Using this statistical approach, and similar physiological system groupings for biomarkers, a study of 1,754 volunteers also reported multisystem dysregulation during frailty (353) and also concluded that no individual systems were more important than others. This is particularly relevant given that the study assessed a group of physiological systems different from that used by Fried et al. (279). However, some noteworthy discrepancies can be seen between these two studies. First, the nonlinearity effect of enhanced frailty risk with an increasing number of dysregulated systems reported by Fried et al. (279) was not corroborated and was attributed to the limited sample size of frail individuals (353). Second, this study did not confirm that the number of systems dysregulated was predictive of frailty presence. This inconsistency may be partially explained by the different definitions of frailty criteria adopted across studies, which has been shown to affect the agreement and predictive ability of the physical frailty phenotype (354). Furthermore, the sample in Fried et al., (279) comprised all female participants, whereas the cohorts studied by Ghacem et al. (353) included men and women. The widely reported greater prevalence of frailty in females (355) suggests there may be a sex difference in the physiological characteristics of frailty, which may contribute to differential findings across these studies.

Multisystem dysregulation has also been reported by other research groups. With the use of previously established cutoff points, against which measured values for different systems were compared, the prevalence of frailty was found to be directly related to the number of abnormal organ systems (when considering cardiac, vascular, pulmonary, renal, hematologic, and adipose systems) (203). Additionally, this study found that cardiac abnormalities showed the strongest association with frailty compared with the other organ systems measured, supporting the premise outlined above that the heart is a key organ contributing to frailty development.

The observations of multisystem dysregulation support the concept of frailty as a condition of numerous abnormalities in a complex system (i.e., the human body). However, current findings from studies comparing physiological characteristics across systems and organs may be compromised by less precise and inaccurate assessment methodologies. For example, whole body adiposity has been measured with skinfold thickness (279) and BIA methods (203), which are less robust than DEXA and MRI but were likely adopted because of their feasibility of application in studies involving large participant numbers. Furthermore, the physiological systems assessed in many studies are distinguished based on circulating biomarkers, which are by their very nature likely to be less representative of the associated organ and tissue functions. Thus, to further understand the contribution of different physiological systems to the frailty phenotype and to more accurately model and predict frailty progression, future studies should strive to gather more direct measures of key organ structure and function to expand on initial circulating biomarker-based reports.

### 3.2. The Physiological Phenotype of Frailty: Using a Stress Stimulus Paradigm

The literature described thus far has identified numerous physiological traits associated with frailty. Despite this, the distinct physiological characteristics of frailty remain poorly understood. This lack of clarity may be because many studies are performed under resting-state conditions, thus failing to capture the dysregulation of dynamic homeostasis that is central to the definition of frailty (356). In short, in the absence of acute infection, illness, and injury, without the presence of external stressors such as physical activity, the dysregulation of physiological homeostasis in frailty may be subtle or undetectable, particularly in the absence of robust and sensitive measurement techniques to quantify physiological resilience. Thus, the phenotypic traits of frailty would likely manifest more overtly than in the resting state if individuals were studied during a physiological stress challenge, such as exercise (FIGURE 7), particularly if using state-of-the-art dynamic measurement approaches to quantify physiological responses. Indeed, frailty is considered as a state during which an individual’s ability to cope with and combat stressors is reduced (8), i.e., reduced resilience. Accordingly, the measurement of dynamic responsiveness to physiological stressors has been identified as a fundamental next step in frailty research (357). Despite this, understanding of the physiological responses to stressors during frailty remains limited, with much less available data relative to measures made in the resting state (outlined above). Nonetheless, a recent review by Fried and colleagues (358) discussed various physiological responses to stressors during frailty, which, promisingly, indicates that this area of research is gaining attention. This section attempts to summarize the current evidence and understanding of the physiological responses to stressors during frailty.

A highly effective method of inducing physiological stress in vivo is acute exercise. A bout of exercise will induce rapid and marked changes in physiological function involving multiple organs (for review see Ref. 359). For example, FIGURE 7 illustrates the change in cardiac output and its distribution transitioning from rest to vigorous exercise across multiple organ systems.

#### 3.2.1. Skeletal muscle energy metabolism.

**FIGURE 9.**
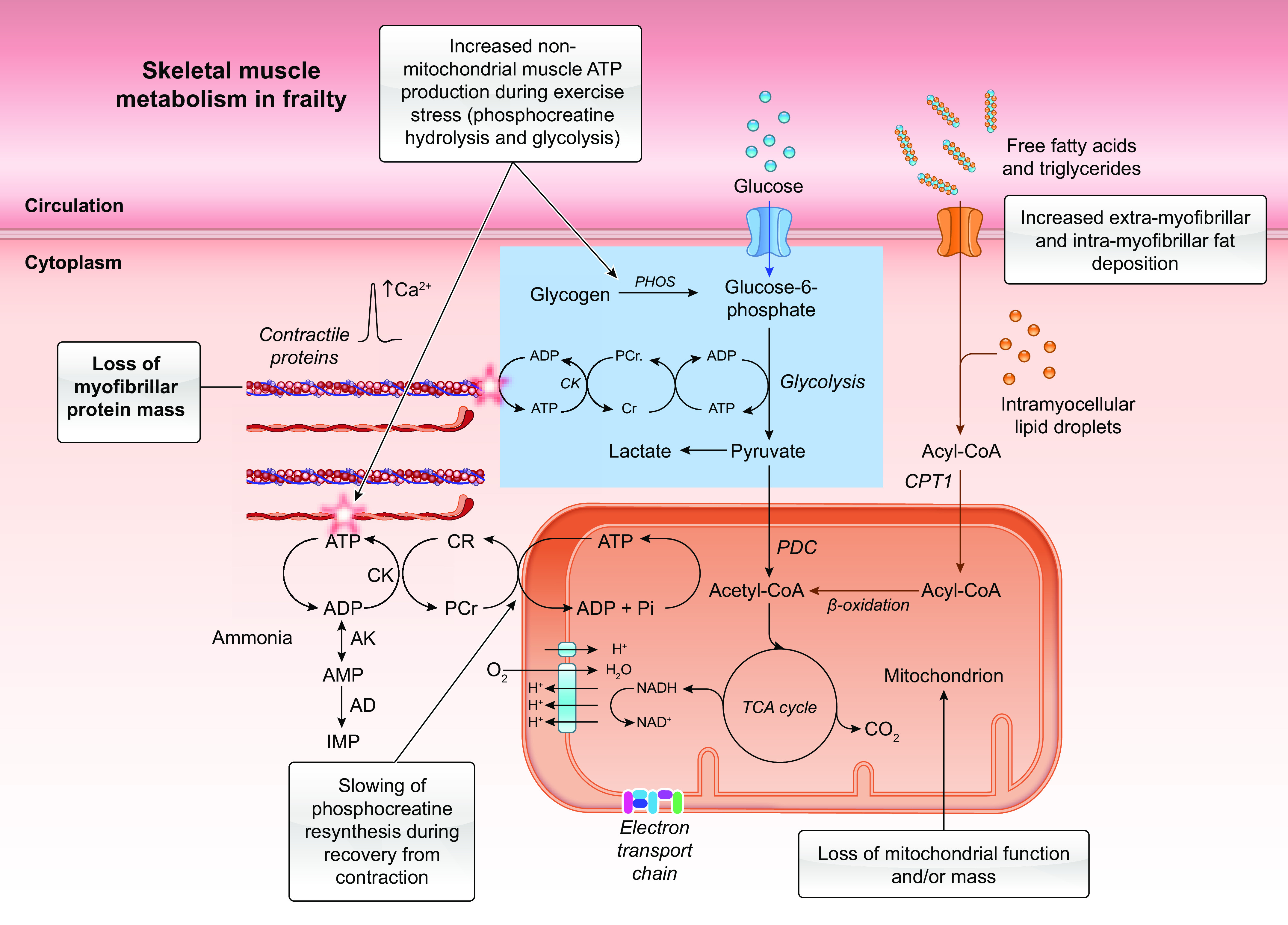
Schematic illustration of the effect of frailty on substrates and pathways involved in skeletal muscle energy turnover. When the rate of ATP demand during muscle contraction exceeds that of mitochondrial ATP production, ATP turnover is maintained from nonmitochondrial routes, namely glycolysis and phosphocreatine (PCr) hydrolysis. ADP, adenosine diphosphate; AMP, adenosine monophosphate; ATP, adenosine triphosphate; Ca^2+^, calcium; CK, creatine kinase; CPT1, carnitine palmitoyltransferase I; Cr, creatine; H^+^, hydrogen ion; H_2_O, water; IMP, inosine monophosphate; NAD^+^, oxidized nicotinamide adenine dinucleotide; NADH, reduced nicotinamide adenine dinucleotide; PCr, phosphocreatine; PDC, pyruvate dehydrogenase complex; Pi, inorganic phosphate; TCA cycle, tricarboxylic acid cycle.

^31^Phosphorus magnetic resonance spectroscopy (MRS) represents a robust, noninvasive in vivo approach to quantify muscle PCr and pH changes during exercise and recovery, making it well suited to study age- and frailty-related decline. A recent study employing this approach in age-matched nonfrail and frail older individuals who performed graded multistage plantar flexion exercise within the bore of a 3-T magnet using ^31^P MRS focused on the gastrocnemius and soleus muscles of the calf (65). During exercise, muscle PCr hydrolysis was fourfold greater in the frail participants (and 10-fold greater than middle-aged control participants) when normalized to the work of activity performed. Furthermore, this increased rate of PCr hydrolysis was strongly inversely associated with performance on a 6-min walk test and peak oxygen uptake (65). These results help illuminate potential physiological mechanisms underpinning the reduced physical function and subjective sense of fatigue in frailty (16). Of interest, this study also reported no difference in MRI-derived calf muscle CSA when comparing frail and nonfrail individuals. Instead, the muscle CSA fat fraction (expressed as a proportion of total muscle area) of frail individuals was greater than that of their nonfrail counterparts (65). Furthermore, the fat fraction was positively associated with PCr hydrolysis, suggesting that differences in muscle metabolic quality, rather than mass, can differentiate the frail phenotype. It also begs the question as to whether increased habitual physical activity intervention in frail people could improve muscle metabolic resilience and thereby functionality in everyday living.

Exercise necessitates a rapid and sustained increase in muscle ATP turnover, from ∼0.07 mol ATP/min at rest to >2 mol ATP/min in heavy exercise (360). When the rate of ATP demand exceeds that of mitochondrial ATP production, energy is derived from nonmitochondrial routes, namely anaerobic glycolysis and phosphocreatine (PCr) hydrolysis (FIGURE 9). Muscle lactate accumulation and PCr hydrolysis during exercise are robust markers of muscle myopathy (361, 362) and mitochondrial dysfunction (363). Furthermore, muscle deconditioning and mitochondrial loss in aging and chronic disease are associated with increased nonmitochondrial muscle ATP production during exercise stress (38, 364). Finally, as muscle PCr resynthesis following exercise is entirely mitochondrion dependent, the slowing of PCr resynthesis kinetics during recovery from exercise can be viewed as a robust index of mitochondrial function and/or mass (365, 366). Changes in muscle energy metabolism during exercise and recovery are therefore likely to provide valuable insight into muscle metabolic and functional decline during frailty.

Considering exercise recovery, Andreux and colleagues (367) compared calf muscle PCr recovery following plantar flexion exercise in prefrail and nonfrail older individuals using ^31^P MRS at 7 T. Prefrail individuals exhibited longer PCr recovery times than physically active nonfrail counterparts, suggesting that reduced mitochondrial respiration/content is a feature of the prefrail state. However, this study did not report the muscle PCr concentration immediately after exercise, making it difficult to interpret the findings, i.e., was the slower recovery a consequence of differences in the rate of ATP turnover, and thereby PCr degradation, during exercise? Given that cellular ADP concentration is a primary driver of postexercise mitochondrial resynthesis, this is a pivotal question to resolve.

A noteworthy limitation of the work described above concerns the lack of efforts to normalize PCr recovery kinetics to total mitochondrial content across the muscle of interest. Without this normalization, mitochondrial dysfunction cannot be assumed because a lower mitochondrial content would also slow PCr recovery kinetics. Indeed, the available data indicate that dysfunction in mitochondrial respiration that is apparent in aging (38) and chronic disease [e.g., COPD (368), diabetes (369)] fails to persist when mitochondrial respiration is corrected for muscle mitochondrial content. Accordingly, “mitochondrial dysfunction” in older people was reversed by exercise training increasing mitochondrial content (38). Assessing succinate dehydrogenase as a marker, lower mitochondrial content has been observed in prefrail compared with nonfrail men in all fiber types of the vastus lateralis (370). A lower vastus lateralis muscle mitochondrial content has also been demonstrated in prefrail and frail women compared with young inactive participants (371). Additionally, large cohort studies have revealed inverse associations between mitochondrial DNA (mtDNA) copy number (an index of mitochondrial number) and polymorphisms in mtDNA with frailty (372, 373). Furthermore, lower abundance and maximal activity of mitochondrial respiratory complexes has been reported in muscle of frail and prefrail compared with nonfrail individuals (367, 374).

Collectively, these findings point to greater research being needed to differentiate between the relative contribution of mitochondrial dysfunction and decline in mitochondrial content to the loss of metabolic resilience in frailty. However, irrespective of this point, emerging evidence indicates that altered muscle energy metabolism is a key underlying feature of generalized physiological decline and fatigue in frailty (FIGURE 9). Furthermore, as the change in tissue energy metabolism is seemingly associated with dysregulation across numerous different organ systems, this may be a common biological feature of frailty-related decline.

#### 3.2.2. Responses to feeding.

Alternative to exercise stress, a substantial physiological response can also be elicited by feeding. After ingestion of carbohydrates, plasma glucose concentrations increase, stimulating pancreatic insulin secretion. Insulin facilitates skeletal muscle and hepatic glucose uptake for storage and/or use; thus, insulin secretion and action are key responses mediating glucose tolerance. Aging is associated with changes in the response to feeding, with older adults demonstrating decreased insulin sensitivity and elevated blood glucose levels after an oral glucose challenge (375, 376). Although many studies have demonstrated insulin resistance in healthy older participants, fewer studies have controlled for typical physiological characteristics of aging that may influence the interpretation of results, such as muscle mass, a decline in habitual physical activity, changes in liver size, and delays in gut carbohydrate absorption. These limitations make it difficult to infer whether impaired glucose tolerance is a feature of normal aging per se or a consequence of age-related changes in lifestyle factors that vary in presence and magnitude between individuals.

An oral glucose tolerance test (OGTT) has been used to elicit a physiological response across different frailty states. Kalyani and colleagues (377) reported no differences in fasted blood glucose and insulin concentrations between frailty states. However, after an oral glucose challenge frail females exhibited exaggerated increases in blood glucose and insulin concentrations over 180 min compared with prefrail and nonfrail women, demonstrating impaired glucose tolerance (377). These findings are consistent with the observation that plasma glucose concentration was elevated 2 h after oral glucose ingestion in frail volunteers compared with nonfrail individuals but not in the baseline fasted state (343). Similarly, after a standardized 700-kcal liquid mixed-meal test, the area under the curve values for 5 h after consumption for glucose and insulin were elevated in frail compared with nonfrail women (378). Although these findings may reinforce an apparent reduction in glucose tolerance in frail individuals, frailty in this study was defined using only the slow gait speed and low physical activity criteria of the physical frailty phenotype (16) and thus may be deemed an inappropriate evaluation of frailty ascertainment. That said, there is evidence that these two frailty criteria are the most predictive components of the frailty phenotype assessment (379), potentially supporting the assessment of frailty in this way.

The studies outlined above suggest that glucose tolerance is impaired during frailty. However, nutrient absorption in the gastrointestinal tract often deteriorates with age (380) and therefore will influence glucose absorption after an OGTT or meal test. Furthermore, body size will influence the blood glucose response when a fixed dose of carbohydrate is administered, e.g., in the OGTT. For this reason, researchers may employ an intravenous glucose tolerance test or the euglycemic insulin clamp technique to control for the effects of gut absorption and body size/lean mass on blood glucose disposal (and insulin action in the case of the insulin clamp technique). When this has been done, the rate of glucose disposal normalized to body surface area (and across a range of steady-state insulin infusion rates) was less in healthy, nonobese older volunteers compared with younger volunteers (381). The same is true when comparing older lean and obese individuals at the level of whole body and leg glucose uptake (346). Although equivalent data in frail volunteers are missing, these lower rates of normalized whole body and leg glucose disposal in older versus young people demonstrate that insulin resistance with age is a real phenomenon, and likely to be multifactorial. It appears that methods such as the quantitative insulin sensitivity check index and homeostasis model assessment scores have been most frequently adopted to assess insulin sensitivity in frailty (382–384). However, these approaches are estimates based on fasting blood glucose and insulin concentration and therefore do not reflect the dynamic gluco-regulatory response to feeding. Accordingly, in the Baltimore Longitudinal Study of Aging, glucose level at 2 h after OGTT was a better predictor of mortality risk than fasting glucose alone (376, 385), with similar findings evident in the Cardiovascular Health Study concerning incident cardiovascular events (386). Although not specific to frailty, these findings reinforce the importance and efficacy of studying physiological characteristics under conditions of stress in order to effectively interpret results.

## 4. EXERCISE INTERVENTIONS IN FRAILTY PREVENTION

In the last 10 years there has been a noticeable increase in exercise-based interventions to limit, reverse, or prevent frailty in older adults (TABLE 2). This is because it is becoming increasingly recognized that regular exercise induces positive adaptation in most, if not all, organ/physiological systems. As described above, muscle weakness, low physical activity, and slowness are the most discriminant physical components of frailty, suggesting that they are important modifiable targets for interventions (401–403). As such, multifactorial interventions (e.g., nutrition, psychosocial, and balance) that include increased exposure to exercise are strong candidates for targeting components of frailty (404). Several meta-analyses have examined the strength and outcomes of exercise trials that aim to change frailty status or reduce frailty prevalence (405–410) (TABLE 2). Although there is heterogeneity among trials, those that include exercise interventions generally favor better outcomes over non-exercise-based interventions (408). Reasons for such variance are the heterogeneity of study design and study populations. In general, the study populations are also multimorbid, with many participants having 10 or more chronic diseases (408). Additionally, although several studies have assessed the impact of exercise interventions on individual components of frailty in nonfrail older adults (e.g., walk speed and grip strength) and observed positive effects, results require careful interpretation (408, 409). Specifically, as frailty is a complex construct, focusing effects on one dimension of frailty may not adequately address an individual’s underlying drivers of frailty. In this section, we review the findings of exercise interventions that have determined changes specifically on frailty in prefrail or frail older adults (TABLE 2). We discuss the components of frailty that were changed by exercise interventions and attempt to link findings to pathophysiological drivers of frailty.

**Table 2.** Large cohort exercise intervention studies to reduce frailty

		Frailty		Intervention				Effects on Frailty	
Population	*N* (% female); Age, yr (mean ± SD)	Measure	Baseline Prevalance	Study groups	Exercise prescription	Duration + follow-up	Aligned with activity guidelines^c^		
*Frail only*									
Kim et al. 2015 (RCT) (387)	131 (100%); 80.9 ± 2.9	Fried frailty	Frail (100%); mean score = 3.7 ± 0.7	*1*) Control (dietary placebo)	2 × week	3 mo + 4 mo follow-up	No (no specified aerobic)	Frailty reclassified (3 mo)	
				*2*) Dietary supplement (MFGM)	60-min/session			*1*) 30.3%	MFGM + Ex > Placebo & MFGM alone
				*3*) MFGM + exercise training	Moderate intensity			*2*) 28.1%	
				*4*) Placebo + exercise training	Strengthening, balance, gait			*3*) 57.6%*	
					Supervised			*4*) 51.5%	
								Frailty reclassified (follow-up)	MFGM + Ex & Placebo + Ex > Placebo
								*1*) 15.2%	
								*2*) 25.0%	
								*3*) 45.5%*	
								*4*) 39.4%*	
Tarazona-Santabalbina et al. 2016 (RCT) (388)	100 (54%); 80.0 ± 3.7	Fried frailty	Frail (100%); mean score = 3.7 ± 0.7	*1*) Exercise	5 × week	24 wk	Yes	Frailty reclassified	Ex > Control
				*2*) Control	65-min/session			*1*) 31.4%*	
					Proprioception & balance			*2*) 0	
					Aerobic & strength				
					Stretching				
Cameron et al. 2013 (RCT) (389)	216 (68%); 83.3 ± 5.9	Fried frailty	Frail (100%); mean score = 3.4 ± 0.7	*1*) Multifactorial and frailty specific	10 × supervised sessions and WEBB^a^ recommendations (balance, strength, aerobic)	12 mo	No (no specified aerobic)	Frailty reclassified	Intervention > Control
				*2*) Control				*1*) 38%*	
								*2*) 24%	
Cesari et al. 2015 (RCT) (390)	424 (68.9%); 76.8 ± 4.2	Fried frailty	Unclear but assumed to be between 20% and 25% considered frail at baseline	*1*) Physical activity	3 × supervised week (*weeks 1–8*)	12 mo	Yes	Prevalance of frailty	Intervention < Controls
				*2*) Health education (Control)	2 × supervised week (*weeks 9–24*) + 3 × home based			*1*) 10%*	
					Home based after *week 2*5			*2*) 19.1%	
					Walking, flexibility, strength				
*Prefrail only*									
Serra-Prat et al. 2017 (RCT) (391)	172 (56.4%); 78.3 ± 4.9	Fried frailty	Prefrail (100%); mean score = 1.45 ± 0.5	*1*) Intervention	Aerobic exercise: 4 × week, 30-45 min/session, walking, home-basedStrength & balance: 4 × week, 20-25 min/session, progressive, home-based	12 mo	Yes	Frail vs. nonfrail:	Intervention < Control
				*2*) Control				*1*) 4.9%*	
								*2*) 15.3%	
								Robust vs. nonrobust:	
								*1*) 15.3%	
								*2*) 21.3%	
Chen et al. 2019 (RCT) (392)	70 (65%); 76.1 ± 5.6	Fried Frailty	Prefrail (100%).	*1*) Exercise	3 × week	8 wk	No (No specified aerobic)	Frailty reclassified	Intervention > Control
				2) Control	45–60 min/session			*1*) 81.8%*	
					Elastic band resistance			*2*) 9.1% + 1 person becoming frail	
*Mixed frailty*									
Ng et al. 2015 (RCT) (393)	246 (61.4%); 70.0 ± 4.7	Fried frailty	Prefrail (72%) and frail (28%); mean score = 2.0 ± 0.8	*1*) Usual care control	2 × week	6 mo + 6-mo follow-up	Yes	Frailty reclassified (12 mo)	Each intervention > Control
				*2*) Cognitive training	90-min/session			*1*) 15.2%	
				*3*) Nutritional supplements	Moderate intensity			*2*) 35.6%*	
				*4*) Physical training	Strengthening & balance.			*3*) 35.6%*	
				*5*) Combination treatment	Supervised (1st 3 mo)			*4*) 41.3%*	
					Home-based (2nd 3 mo)			*5*) 47.8%*	
Chan et al. 2012 (Pilot RCT) (394)	117 (59%); 71.4 ± 3.7	Fried frailty	Prefrail (87%) and frail (13%).	*1*) Exercise + nutrition	3 × week	3 mo + 6-, 9-, 12-mo follow-up	Yes	Frailty reclassified (3 mo)	Ex + nutrition > Control of *1*
				*2*) Problem solving therapy	60-min/session			*1*) 45%*	
				*3*) Control of *1*	Brisk walking, stretching, strengthening, balance			*2*) 44%	
				*4*) Control of *2*	Supervised			*3*) 27%	
								*4*) 28%	
Seino et al. 2017 (RCT–CO) (395)	77 (31.2%); 74.6 ± 5.5	Completed the HCS + CL15 frailty score ≥ 2	Prefrail (72.7%) and frail (27.3%); mean score = 3 ± 1.4	Exercise + nutritional + psychosocial	2 × week	3 mo + 3-mo control	No (No specified aerobic)	Intervention reduced CL15 scores that continued during 3-mo postintervention control. Intervention reclassified fralty to prefrailty in 45–58% of frail participants.	Intervention > Controls
				*1*) Immediate	60-min/session				
				*2*) Delayed (3 mo)	Resistance program				
Nagai et al. 2018 (RCT) (396)	41 (90.5%); 81.5 ± 7.2	Fried frailty	Prefrail (41.5%) and frail (58.5%)	*1*) Exercise	2 × week	24 wk	Similar (focused on resistance and gave guidance for physical activity)	Frailty reclassified	No difference
				*2*) Exercise + guidance	Resistance training			*1*) 15%	
								*2*) 28.6%	
Chan et al. 2017 (RCT) (397)	289 (53%); 71.6 ± 4.3	Fried frailty	Prefrail (79%) and frail (21%).	*1*) Control (education)	48 sessions	6 mo + 3- and 12-mo follow-up	Yes	Frailty reclassified (6 mo):	No difference
				*2*) Intervention (exerecise + problem solving)	60-min/session			*1*) 39%	
					Brisk walking, stretching, resistance, balance			*2*) 42%	
								Frailty reclassified (12 mo):	
								*1*) 36%	
								*2*) 42%	
Luger et al. 2016 (RCT) (398)	80 (84%); 82.8 ± 8.0	Fried frailty	Robust (1%), Prefrail (35%), frail (64%)	*1*) Exercise + nutrition	2 × week	12 wk	No (No specified aerobic)	Frailty reclassified	No difference
				*2*) Social support	60 min/session			*1*) 17%	
					Muscle strengthening			*2*) 16%	
Oh et al. 2021 (nonrandomized control) (399)	383 (72%)	Fried frailty phenotype and deficit accumulation index	Unclear	*1*) Multicomponent	2 × week	24 wk + 6-, 18-mo follow up	Similar (similar strengthening but less aerobic)	The intervention reduced frailty index and phenotype scores after intervention. Differences were not maintained at future assessments.	Intervention > Controls
	234 (75%)^b^; 76.3 ± 5.7^b^		2.2 ± 1.2 phenotype^b^	*2*) Comparison	60 min/session				
					Resistance (20 min)				
			0.26 ± 0.11 index^b^		Balance (20 min)				
					Aerobic (20 min)				

^a^Weight-bearing for better balance program (WEBB) (400). ^b^After propensity matching. ^c^Alignment with physical activity guidelines for older adults. *P < 0.05, significantly different from control group. CL15, Check-List 15; HCS, Hatoyama Cohort Study; RCT, randomized control trial; RCT-CO, RCT-crossover. See glossary for other abbreviations.

### 4.1. Reversing Frailty in Frail Adults

Before the Fried physical frailty phenotype, one of the most impressive interventions showing positive results in long-term nursing home men and women was the Boston FICSIT study (37, 39). Although frailty was less well defined, the majority of participants were likely frail as shown by low mobility, strength, and nutritional intake measurements. In the first of these studies, 8 wk of high-intensity (∼80% of 1 repetition maximum) supervised progressive lower body resistance training resulted in significant muscle strength, mass, and function gains (39). In the randomized control follow-up study, 10 wk of the same exercise program with or without a dietary supplement also increased muscle strength, mass, and function (37). Together, the Boston FICSIT suggested that high-intensity supervised resistance training could improve physical function in predominantly frail or dysfunctional very old adults.

Given that there were few adverse events and the intervention was feasible, the results of the below trials using predominantly moderate-intensity exercise highlight a continuing debate. Can a frail person perform, and should we expect them to perform, exercise at the necessary intensity and duration to induce frailty improvements? To the best of our knowledge, only three adequately powered and randomized control studies (387–389) and one randomized substudy (390) have been conducted specifically in frail adults with the aim of reversing frailty. With the Fried frailty phenotype, frailty reversal was considered if status changed from frail (score ≥ 3) to either prefrail (score = 1–2) or nonfrail (score = 0) at postintervention and/or follow-up.

Kim et al. (387) assessed 131 women randomized to one of four 3-mo interventions followed by a 4-mo postintervention follow-up. Groups consisted of combinations of either a milk-based nutritional supplement (MFGM) or placebo and twice weekly 60-min moderate-intensity instructor-led exercise classes that included 30 min of strengthening exercises and 20 min of balance and gait training. At the 3 mo time point, between 28.1% and 57.6% of participants were reclassified as not frail, with the exercise and nutritional supplement observing the largest changes in frailty scores. At the 4 mo follow-up, both exercise groups continued to have significantly more reclassified participants than the placebo group, suggesting a positive longevity effect of exercise. Although weight loss, exhaustion, low physical activity, and slow walk speed were improved by exercise, muscle strength and mass were unchanged. Even though the strengthening exercises included arm, leg, and upper body exercises, it is unclear whether this lack of changes resulted from inadequate amounts or intensity of exercise. The Boston FICSIT study clearly shows that increases in muscle mass and strength can be achieved in poorly functiolder adults if the right exercise intervention is used and that in healthy community-dwelling older adults exercise training can increase muscle mass and strength in interventions as short as 3 mo (411).

In an attempt to understand the physiological mechanisms responsible for the improvements seen, Kim et al. measured blood biomarkers associated with general muscle health and brain function. BDNF increased in all groups, indicating that frailty improvements are associated partially with improved neurocognitive capabilities, and other studies have shown that exercise can increase BDNF and neurocognitive functions in healthy older adults (412). Additionally, only the exercise + MFGM group observed reduced myostatin and ratio of IGFBP3 after intervention. Although this would indicate improved muscle health that perhaps contributes to the reduction in frailty, the lack of strength and lean mass changes do not support this. As the IGFBP3/IGF-1 is presented as a ratio, understanding these directional changes is more complex, as it would be expected that lower myostatin and higher IGF-1 would increase muscle mass (413). Myostatin is a negative regulator whereas IGF-1 is a positive regulator of muscle mass, and levels of these blood biomarkers are associated with frailty (82). However, inconsistent group findings for myostatin and IGFBP3/IGF-1 in this study make it challenging to determine the relevance of the results.

Although these results provide evidence that exercise training can reverse frailty in some frail adults, it is unclear why the effects were not observed in all participants. One possible explanation is that the exercise program was not specific for each physical dysfunction that contributed to frailty. To address issue, Cameron et al. (389) assessed 216 men and women randomized to either 12 mo of usual care or a frailty criteria-specific multifactorial intervention. The intervention focused on each participant’s deficit in individual components of frailty. For example, if the weight loss criterion was identified, participants were referred to the study dietician for appropriate nutritional recommendations. The exercise component was prescribed if participants met weakness, slowness, and/or low energy expenditure requirements. The exercise program consisted of 10 home-based physiotherapist sessions and an individualized home-based program that focuses on balance, strengthening, and aerobic exercises using progressive moderate intensities (400).

There were significantly more participants in the exercise group after the intervention than control participants who were no longer frail, although the proportion with reversal of frailty was lower than seen by Kim et al. Similar to Kim et al., there were no differences in muscle strength. Cameron et al. also measured the short physical performance battery and observed improved balance, chair stand, and walk scores at 12 mo, suggesting that muscle health was improving. In most other settings, supervised exercise training is superior to home-based training for positive changes in outcomes and may be so in frail adults. Furthermore, only 44% of participants completed the intervention with >50% adherence (414), with greater adherence associated with better frailty outcomes, suggesting that the amount of exercise needed to see meaningful effects is critical.

In a third study, Tarazona-Santabalbina et al. (388) assessed 100 men and women randomized to either 6 mo of usual care or a multicomponent exercise program (MEP). The MEP consisted of 5 × 65-min group sessions per week, combining short periods of proprioception and balance, low-to-moderate intensities of aerobic exercise, and muscle-strengthening exercises. More MEP participants were no longer classified as frail after the intervention, whereas all control participants remained frail. However, it is unclear from the study which frailty criteria were reduced. Instead, improvements were observed for functional measures, including walk speed and physical performance test, and also cognitive function as measured by the mini-mental state exam (MMSE). Again no changes were observed for lean mass, although lean mass was reported as a percentage and not absolute values, limiting our interpretation of the intervention.

Finally, Cesari et al. conducted exploratory analyses from the Lifestyle Interventions and Independence for Elders pilot (LIFE-P) study (390, 415). Here, 424 community-dwelling men and women were randomized to either 12 mo of successful aging education (control participants) or a progressive physical activity intervention consisting of supervised and home-based activities. At 12 mo, the intervention group was over twice less likely to be frail than control participants. Furthermore, in this paper no indications of physiological measures were given, limiting our ability to relate the study to others, other than a reduction in the incidence of frailty. However, the LIFE-P study was not designed to prevent or reduce frailty, and not all the participants were frail. Therefore, it is likely that this study design was inappropriate for targeting frailty. It is important to note that it is a limitation of such large-scale intervention studies that they rarely include well-controlled exercise protocols, for practical reasons, and moreover the end point measures do not give mechanistic insight.

### 4.2. Lowering the Progression to Frailty in Prefrail Adults

Specifically targeting prefrail adults has the potential to slow down or prevent progression to frailty and adverse frailty outcomes. We are aware of only two large randomized control studies that assessed the prevalence of frailty specifically in adults who were prefrail at baseline (391, 392) (TABLE 2). Serra-Prat et al. (391) assessed 172 men and women classified as prefrail and randomized to either 12 mo of usual care or a nutritional and exercise intervention. Only those at risk of malnutrition were referred to clinical nutritional care, whereas everyone was assigned the exercise program. At 12 mo, the intervention group had fewer participants who had progressed to becoming frail, compared with the control group. No measures of lean mass were performed, and BMI was similar between groups at 12 mo.

More recently, Chen et al. (392) assessed 70 men and women who were randomized to either 8 wk of usual care or an exercise intervention consisting of three weekly supervised sessions of 45–60 min/session of elastic band strengthening exercises. After 8 wk, the intervention group had more participants who were no longer prefrail compared with the control group. No measures of lean mass were performed. Interestingly, the intervention group improved absolute grip strength, walking speed, and physical activity levels. Unlike the aforementioned studies, the increased grip strength was unique and suggests that muscle health can be targeted and improved.

That said, Chen et al., like Serra-Prat et al., targeted grip strength and improved it, suggesting that in prefrail adults targeting one major frailty criterion is enough to reduce the progression of frailty.

These and the frailty-only studies would suggest that exercise training can slow frailty development in prefrail adults while reversing frailty in frail adults and that an intensive supervised group program rather than unsupervised home-based exercise is associated with better improvements in frailty status in prefrail adults.

### 4.3. Interventions in Mixed-Frailty Populations

The previous studies suggest differential responses to exercise depending on the program’s duration and intensity, supervision, and the severity of the frailty classification (i.e., prefrail vs. frail). To date, most randomized studies have assessed the effects of an intervention in a mixed group of frail and prefrail older adults. As a result, the findings are inconsistent because of the heterogeneity of people within the study and the type and duration of interventions.

One of the most comprehensive interventions observed significant reductions in frailty scores and reclassification of frailty status across each intervention group (393). Reclassification was considered if participants changed from frail to prefrail, frail to nonfrail, or prefrail to nonfrail. Ng et al. (393) assessed 246 mostly prefrail and frail men and women randomized to one of five 6-mo interventions and a 6 mo follow-up. Interventions were *1*) usual care with a placebo supplement; *2*) a nutritional supplement; *3*) cognitive training; *4*) exercise training; or *5*) a combination of the nutritional supplement, cognitive, and exercise training. At 6 mo, frailty composite scores were lower in both exercise training groups compared to control participants. At 12 mo, frailty was significantly reclassified in all the groups except the control group, with both exercise groups having the most likelihood of changing their frailty status.

Unlike the studies that used grip strength, compared with control participants the frailty criteria of strength improved for the exercise and combined groups. Although Ng et al. used leg strength as a muscle weakness indicator, which may have biased frailty outcomes, it reinforces our suggestion that specificity in measurements limits our ability to interpret physiological changes. Although lean mass was not measured and BMI remained unchanged, all other frailty criteria improved across certain interventions. This study provides evidence that a period of intensive supervised training at the beginning of the intervention provides the best chance of long-term frailty outcomes.

In a second study, Chan et al. (394) randomized 117 adults who were mostly prefrail or frail to 3 mo of either an exercise and nutrition intervention, a problem-solving therapy (PST) intervention, or one of two controls of each intervention. At the end of the study only the exercise group had significantly more participants who had frailty reclassified to a lower status, with 32% of prefrail participants improved to nonfrail and 40% and 20% of frail participants improved to prefrail and nonfrail, respectively. These data suggest that exercise may equally improve frailty status across differing frailty definitions. However, in terms of the physiological responses, fat-free mass decreased, leg strength increased, but no neurocognitive functions were changed in any of the groups. The frailty criteria used were a modified Fried phenotype with a classification status based on comorbidities. The actual number of comorbidities was relatively low across the groups (average of 3.5 each), and as such the participants were a relatively “healthy” cohort of frail and prefrail participants.

Similarly, Seino et al. (395) used a frailty index designed and validated by themselves and recruited 77 men and women in a randomized 3-mo immediate-start or delayed-start crossover design. The Check-List 15 (CL15) criteria (416, 417) identified 56 participants as prefrail and 21 as frail. Similar to Ng et al. (393), the intervention consisted of exercise, nutritional, and psychosocial guidance. For all participants, regardless of when the intervention started, it reduced frailty scores: 18.4% (immediate) and 12.8% (delayed) of frail participants improved to prefrail or nonfrail, respectively. Similar to Kim et al. (387), there was a legacy effect at the 6 mo follow-up. In terms of physiological responses, although lean mass was not assessed, the intervention increased weight and BMI and improved timed-up-and-go (TUG). At the same time, grip strength was ambiguous and cognitive function remained unchanged. As such, it is difficult to determine which physiological improvements were driving lowered frailty scores and increased reclassification in frailty. Taken together, the three studies above suggest that exercise training may equally lower frailty scores and status in frail and prefrail older adults, with frail adults more likely to improve status.

We identified three trials with no effects compared to control participants. Nagai et al. (396) assessed whether the addition of aerobic exercises to a resistance training program would improve frailty. With both groups receiving resistance training, the 24-wk study in 41 frail and prefrail men and women observed reduced frailty scores in those with the addition of aerobic training. However, this did not translate to significant differences between groups for frailty classification. The combined group improved the frailty criteria for weight loss and grip strength, whereas the exhaustion criteria worsened in the control group. In terms of physiological changes, the combined group increased leg strength and power, time spent in low-intensity physical activity, and cognitive behavior changed more than in the control participants. Both groups equally improved their walking speed and TUG times. These effects suggest that resistance plus aerobic training for 24 wk can improve muscle strength, components of cardiovascular fitness, and cognitive function more than resistance, whereas physical performance is equally improved with resistance training.

Chan et al. (397) completed the follow-up to their 2012 pilot study (reviewed above in this section) and utilized similar intervention components, except combined into one intervention with two groups. Here, they assessed 289 mainly prefrail and frail men and women randomized to 6 mo of either a predominantly home-based DVD or an intensive supervised exercise and problem-solving sessions and the home DVD. At 6 mo, with ∼40% of all participants changing frailty status, both groups observed similar effects between home-based and supervised interventions. With the modified frailty index that reflected the Taiwanese population, at most time points frailty criteria improvements were observed for exhaustion, energy expenditure, 5-m walking time, and grip strength. Although these modified frailty scores were improved, only the TUG and one-leg-stand time improved, whereas lean mass remained unchanged for the Fried frailty phenotype. As such, both an intensive and a less intensive intervention may improve frailty criteria.

Finally, Luger et al. (398) assessed 80 mostly prefrail and frail men and women randomized to 12 wk of either social support (control group) or a whole body resistance-based exercise and nutrition intervention. After 12 wk, both groups combined significantly reduced the prevalence of frailty, but no differences between groups were observed. This study focused on nutritional health, and as such no measures of individual frailty criteria or muscle mass were completed, limiting our ability to determine physiological responses.

### 4.4. Longevity of the Impact of Interventions

A final aspect of interventions is the longevity, or legacy, of the observed effect. Few studies have considered this element, but recently Oh et al. (399) reported on a nonrandomized multicomponent intervention in 383 socioeconomically vulnerable older Korean men and women. One hundred eighty-seven participants chose the 6-mo intervention consisting of supervised group exercise sessions. In addition, participants received a daily nutritional supplement, medication assessment to reduce polypharmacy, therapy for depression if this was diagnosed, and home environment assessment to minimize trip hazards. Frailty was assessed by the Fried frailty phenotype and the deficit-accumulation frailty index at baseline (6 mo before the start of the intervention) and at the end of the intervention, plus 6 mo after the intervention completion and again 12 mo later. The baseline scores for frailty phenotype and frailty index suggest that the groups were largely prefrail. The intervention group were frailer, suggesting that less frail individuals are less likely to desire an intervention. At the end of the 6-mo intervention, the intervention group had a lower frailty index and phenotype scores than control participants. However, when participants were reassessed 6 and 18 mo after the intervention, the differences between groups were nonsignificant. Nevertheless, at the end of the intervention, the intervention group had significantly higher physical performance scores (SPPB), and these scores remained higher than control participants until the completion of the study 18 mo later. As such, these findings are in line with other studies in prefrail adults but critically suggest that interventions must be maintained for the benefit to persist, which is to be expected.

### 4.5. Summary Exercise Interventions in Frailty Prevention

Taken together, when exercise is included as part of a frailty prevention or reduction program, positive effects compared with usual care control groups are generally observed. Specifically, if exercise is part of a multimodal approach that also targets other components of frailty, including nutritional deficits, psychosocial education, or cognitive function, effects are larger and appear more robust over time. Frailty scores and frailty status appear to be improved more when the program is designed for frailty, rather than other conditions such as poor mobility. Additionally, adherence is often low and may explain, in part, the heterogeneity of responses. Increasing adherence, either through simplifying the program or conducting it in a supervised environment, will likely improve outcomes. However, not all supervised interventions improved frailty status. We noticed that the majority of studies prescribe exercise using nonspecific, often qualitative physiological measures, including rating of perceived exertion (RPE) or predicted maximum heart rate. Although this approach is more generalizable, it often over- or underestimates exercise intensity, making it challenging to compare results and determine possible underlying physiological mechanisms. For example, we observed that there is mostly a lack of effect of exercise on individual frailty criteria, muscle mass, and muscle strength. Nonfrail older adults typically respond more positively to exercise training studies prescribed from exact fitness measures. However, from the current literature, it is unclear whether the lack of effects on muscle results from too low exercise intensities caused by nonspecific prescription or an effect from the underlying pathophysiological causes of an individual’s frailty. The work from the Boston FICSIT Study would suggest that it may be too low exercise intensities.

## 5. KNOWLEDGE GAPS AND RECOMMENDATIONS FOR FUTURE RESEARCH

Frailty is currently defined by clinical criteria based either on the physical phenotype or the accumulation of deficits, with little assessment of the physiological changes that drive the criteria. We suggest that this is limiting our ability to adequately stratify prefrail and frail older adults and design targeted interventions to reduce or prevent frailty developing. Importantly from a physiological standpoint, the majority of studies have involved assessment of the characteristics of individual organs and have been carried out under resting-state conditions. This is not optimal for understanding frailty, which is a complex multiorgan condition whose definition is based upon a decline in robustness or resilience to stressors.

### 5.1. Recommendation 1

We suggest that, going forward, we require integrative modeling of individual physiological components at rest and under challenge, including through exercise, to define the physiological phenotype of frailty. In addition to this overarching change in approach to frailty, we suggest that there are distinct gaps in our understanding or approach to frailty research that should be addressed in future research studies:

#### 5.1.1. Clinical.

Clinical studies should focus on reporting the phenotypic differences between nonfrail and frail older individuals so it is clear moving forward what we define as normal or healthy aging, a chronological process that does not affect function, as opposed to unhealthy aging, a pathological process that leads to reduction in function (of a person, physiological system, or organ system). These clinical studies need deliberate matching to concurrent study of the underlying physiology we discuss below.

#### 5.1.2. Brain.

Several aspects of age-related changes to brain anatomy and physiology are underresearched in relation to their contribution to frailty. For example, is frailty per se, or elements of the syndrome’s component criteria, underpinned by reduced brain volumes in specific brain regions? Using a range of brain imaging methods will be important to determine how brain alterations lead to physical presentations. For example, decreased cerebral oxygenation may explain the apparent attenuations in neuromuscular function during frailty (119). Reduced cerebral blood flow and cerebrovascular reactivity have been reported during normal aging (418) and may also present as a feature of the frailty state, potentially contributing to brain structure deterioration during frailty (144).

#### 5.1.3. Skeletal muscle.

There are clear associations between skeletal muscle deficits and frailty, with studies to date suggesting that muscle quality and mass are drivers of poor physical function and weakness seen in frail adults. Further studies are needed to define, for example, the roles of anabolic resistance, increased fat infiltration, insulin resistance, compromised satellite cell function, and reduced NMJ number and function. In relation to mitochondrial function and metabolic resilience in frailty, more research is needed to differentiate between the relative contribution of mitochondrial dysfunction and the decline in mitochondrial content seen in the muscle of frail adults. Whatever the outcome of this research, the current literature indicates that altered muscle metabolism is a key underlying feature of physiological decline and fatigue in frailty.

#### 5.1.4. Study design.

Frailty research to date has mainly involved a single cross-sectional assessment of frailty(420). Some studies have assessed the longitudinal associations between frailty and brain architecture variables, such as WMH volume, microstructural integrity, and macroinfarcts (166, 421, 422). However, interpretation of findings from these studies is restricted by factors such as an inadequate number of frail individuals recruited and prospective study designs incorporating only a single assessment of physiological parameters. Similarly, a small number of studies have attempted to investigate associations between alterations in body composition characteristics and frailty over time. However, this literature is confounded by indirect measures of body composition and skeletal muscle mass (423). These limitations underpin a poor understanding of the temporal relationships between frailty development and underlying physiological changes.

### 5.2. Recommendation 2

To try and understand the factors influencing the trajectory from a nonfrail state to frailty, large and robust longitudinal studies assessing temporal relationships between a broad range of physiological parameters and frailty in the same individuals should be prioritized.

### 5.3. Recommendation 3

Key to elucidating mechanisms of frailty development will be the design and implementation of intervention studies, with for example well-controlled exercise protocols and end point measures, in longitudinal study designs with associated mechanistic analyses.

If specific pathophysiological characteristics and frailty status are improved in tandem by intervention, these physiological processes may be deemed contributing factors to frailty progression. One example in this area is a study using 6 mo of a resistance exercise training program in nonfrail and prefrail older adults and showing improved leg strength in both groups. Transcriptomic analysis of muscle biopsies revealed that the improvement in strength was associated with the protocadherin gamma gene cluster, which may be related to muscle denervation and reinnervation (32).

### 5.4. Recommendation 4

Although inflammation increases with age and is associated with increased risk of frailty in large population-level studies and meta-analyses (263), it is still not clear that there is a causative role of inflammation in the development of frailty. Direct interventional studies in humans assessing the impact on frailty as an end point are required and must progress beyond the current literature, which is largely focused on sarcopenia. We recognize that such studies will not be straightforward, as many frail older adults are already prescribed drugs that will modify their inflammatory status. Furthermore, given the multitissue compromise seen in frailty (e.g., muscle, brain, heart), future studies should consider both local and systemic inflammatory profiles and take a systems modeling approach to understanding the range of influences on frailty at the individual level.

## 6. CONCLUSION

In summary, frailty is a complex multiorgan condition that is currently described in clinical rather than physiological terms. To better understand and treat frailty, we suggest that a multiorgan approach is required, harnessing state-of-art technologies to quantify organ structure and function. Inflammation is associated with frailty development, but proof of causation is lacking. Studies to address this issue may be confounded by the multimorbid, multimedicated nature of many frail adults. On a positive note, there is evidence that interventions that include exercise can reduce and reverse frailty. However, the most successful are delivered in person rather than via remote home-based programs.

## GLOSSARY

ADL: Activities of daily living
ASL: Arterial spin labeling
ATP: Adenosine triphosphate
BAK-1: BCL2 antagonist/killer 1
BDNF: Brain-derived neurotrophic factor
BIA: Bioelectrical impedance analysis
CHS: Cardiovascular Health Study
CMAP: Compound muscle action potential
COPD: Chronic obstructive pulmonary disease
CRP: C reactive protein
CSA: Cross-sectional area
cSVD: Cerebral small vessel disease
CT: Computed tomography
CXCL13: C-X-C motif chemokine ligand 13
DEXA: Dual-energy X-ray absorptiometry
DHEAS: Dehydroepiandrosterone sulfate
DIG: Delayed intervention group
DNA: Deoxyribonucleic acid
DTI: Diffusion tensor imaging
EF: Ejection fraction
EMRA: Effector memory-expressing RA
fMRI: functional MRI
FOXM1: Forkhead box M1
FSR: Fractional synthetic rate
iEMG: Intramuscular electromyography
IFNγ: Interferon gamma
IGF-1: Insulin-like growth factor 1
IGFBP3: Insulin-like growth factor binding protein 3
IL: Interleukin
IMAT: Intramuscular adipose tissue
LCFA: Long-chain fatty acid
MD: Mean diffusivity
MFGM: Milk fat globule membrane complex powder
MRI: Magnetic resonance imaging
MRS: Magnetic resonance spectroscopy
mtDNA: Mitochondria DNA
mTOR: Mammalian target of rapamycin
MU: Motor unit
MUP: Motor unit potential
NF-κB: Nuclear factor kappaB
OGTT: Oral glucose tolerance test
PCr: Phosphocreatine
PST: Problem solving therapy
PUMA: p53-Upregulated modulator of apoptosis
RASM: Relative appendicular skeletal muscle mass
RNA: Ribonucleic acid
SASP: Senescence-associated secretory phenotype
SMA: Supplementary motor area
SNP: Single-nucleotide polymorphism
STAT: Signal transducer and activator of transcription
TNF-α: Tumor necrosis factor-alpha
WMH: White matter hyperintensity

## GRANTS

J. A. Taylor is supported by a PhD scholarship funded by the MRC-Versus Arthritis Centre for Musculoskeletal Ageing Research (MR/P021220/1). J. M. Lord is supported by the NIHR Birmingham Biomedical Research Centre (BRC-1215-20009), and P. L. Greenhaff by the NIHR Nottingham Biomedical Research Centre.

## DISCLAIMERS

The views expressed here are those of the authors and not necessarily those of the National Health Service (NHS), the National Institute for Health and Care Research (NIHR), or the Department of Health and Social Care.

## DISCLOSURES

No conflicts of interest, financial or otherwise, are declared by the authors.

## AUTHOR CONTRIBUTIONS

J.A.T., P.L.G., and D.B.B. prepared figures; J.A.T., P.L.G., D.B.B., T.A.J., N.A.D., and J.M.L. drafted manuscript; J.A.T., P.L.G., D.B.B., T.A.J., N.A.D., and J.M.L. edited and revised manuscript; J.A.T., P.L.G., D.B.B., T.A.J., N.A.D., and J.M.L. approved final version of manuscript.
